# Silencing LCN2 suppresses oral squamous cell carcinoma progression by reducing EGFR signal activation and recycling

**DOI:** 10.1186/s13046-023-02618-z

**Published:** 2023-03-11

**Authors:** Zixian Huang, Xi Rui, Chen Yi, Yongju Chen, Rui Chen, Yancan Liang, Yan Wang, Weicheng Yao, Xiaoding Xu, Zhiquan Huang

**Affiliations:** 1 Department of Oral and Maxillofacial Surgery, Sun Yat-Sen Memorial Hospital, Sun Yat-Sen University, Guangzhou, Guangdong China; 2 Guangdong Provincial Key Laboratory of Malignant Tumor Epigenetics and Gene Regulation, Medical Research Center, Guangdong-Hong Kong Joint Laboratory for RNA Medicine, Sun Yat-Sen Memorial Hospital, Sun Yat-Sen University, Guangzhou, China; 3 Hospital of Stomatology, The First Affiliated Hospital, Jinan University, Guangzhou, China; 4 Nanhai Translational Innovation Center of Precision Immunology, Sun Yat-Sen Memorial Hospital, Foshan, 528200 China; 5 Guanghua School of Stomatology, Hospital of Stomatology, Sun Yat-Sen University, Guangzhou, Guangdong China; 6 Department of Stomatology, Sun Yat-Sen Memorial Hospital, Sun Yat-Sen University, Guangzhou, Guangdong China

**Keywords:** Oral squamous cell carcinoma, Lipocalin-2, EGFR, Metastasis, Proliferation, Nanoparticles

## Abstract

**Background:**

EGFR is an important signal involved in tumor growth that can induce tumor metastasis and drug resistance. Exploring targets for effective EGFR regulation is an important topic in current research and drug development. Inhibiting EGFR can effectively inhibit the progression and lymph node metastasis of oral squamous cell carcinoma (OSCC) because OSCC is a type of cancer with high EGFR expression. However, the problem of EGFR drug resistance is particularly prominent, and identifying a new target for EGFR regulation could reveal an effective strategy.

**Methods:**

We sequenced wild type or EGFR-resistant OSCC cells and samples from OSCC patients with or without lymph node metastasis to find new targets for EGFR regulation to effectively replace the strategy of directly inhibiting EGFR and exert an antitumor effect. We then investigated the effect of LCN2 on OSCC biological abilities in vitro and in vivo through protein expression regulation. Subsequently, we elucidated the regulatory mechanism of LCN2 through mass spectrometry, protein interaction, immunoblotting, and immunofluorescence analyses. As a proof of concept, a reduction-responsive nanoparticle (NP) platform was engineered for effective LCN2 siRNA (siLCN2) delivery, and a tongue orthotopic xenograft model as well as an EGFR-positive patient-derived xenograft (PDX) model were applied to investigate the curative effect of siLCN2.

**Results:**

We identified lipocalin-2 (LCN2), which is upregulated in OSCC metastasis and EGFR resistance. Inhibition of LCN2 expression can effectively inhibit the proliferation and metastasis of OSCC in vitro and in vivo by inhibiting EGFR phosphorylation and downstream signal activation. Mechanistically, LCN2 binds EGFR and enhances the recycling of EGFR, thereby activating the EGFR-MEK-ERK cascade. Inhibition of LCN2 effectively inhibited the activation of EGFR. We translated this finding by systemic delivery of siLCN2 by NPs, which effectively downregulated LCN2 in the tumor tissues, thereby leading to a significant inhibition of the growth and metastasis of xenografts.

**Conclusions:**

This research indicated that targeting LCN2 could be a promising strategy for the treatment of OSCC.

**Supplementary Information:**

The online version contains supplementary material available at 10.1186/s13046-023-02618-z.

## Background

Epidermal growth factor receptor (EGFR) belongs to the tyrosine kinase receptor family and is also known as HER1 or erbB1. It is an important regulatory target that participates in cell regeneration, internal environmental homeostasis, and the occurrence and development of tumors by mediating cell proliferation, migration and differentiation [1]. At present, EGFR-based targeted drugs have been clinically used in antitumor therapy, such as cetuximab, afatinib, and erlotinib [2], prolonging patient survival by 10–20% [3]. Oral squamous cell carcinoma (OSCC) is one of the most common malignant tumors and accounts for 1–2% of all malignant tumors throughout the world [4]. It has a high incidence of invasion to surrounding tissues, thereby leading to poor prognosis [5]. The most common site is the cervical lymph nodes, where the incidence of stage I/II OSCC with cervical metastasis is as high as ~ 42%[6]. Studies have reported that the expression rate of EGFR in OSCC accounts for 36%-100% [7]. The application of anti-EGFR drugs in the treatment of OSCC has achieved good results. However, similar to other types of tumors, the problem of drug resistance is particularly prominent[8], and approximately 80% of responders develop drug resistance [9, 10].

Tumor metastasis and drug resistance directly restrict the prognosis of OSCC patients. There is an emerging need for new targets that can block EGFR and inhibit OSCC metastasis. The key to solving this problem is hidden in the problem. Herein, we sequenced wild type or EGFR-resistant OSCC cells and OSCC patients with or without lymph node metastasis to find new targets for EGFR regulation to effectively replace the strategy of directly inhibiting EGFR and exert an antitumor effect.

**Scheme 1:**
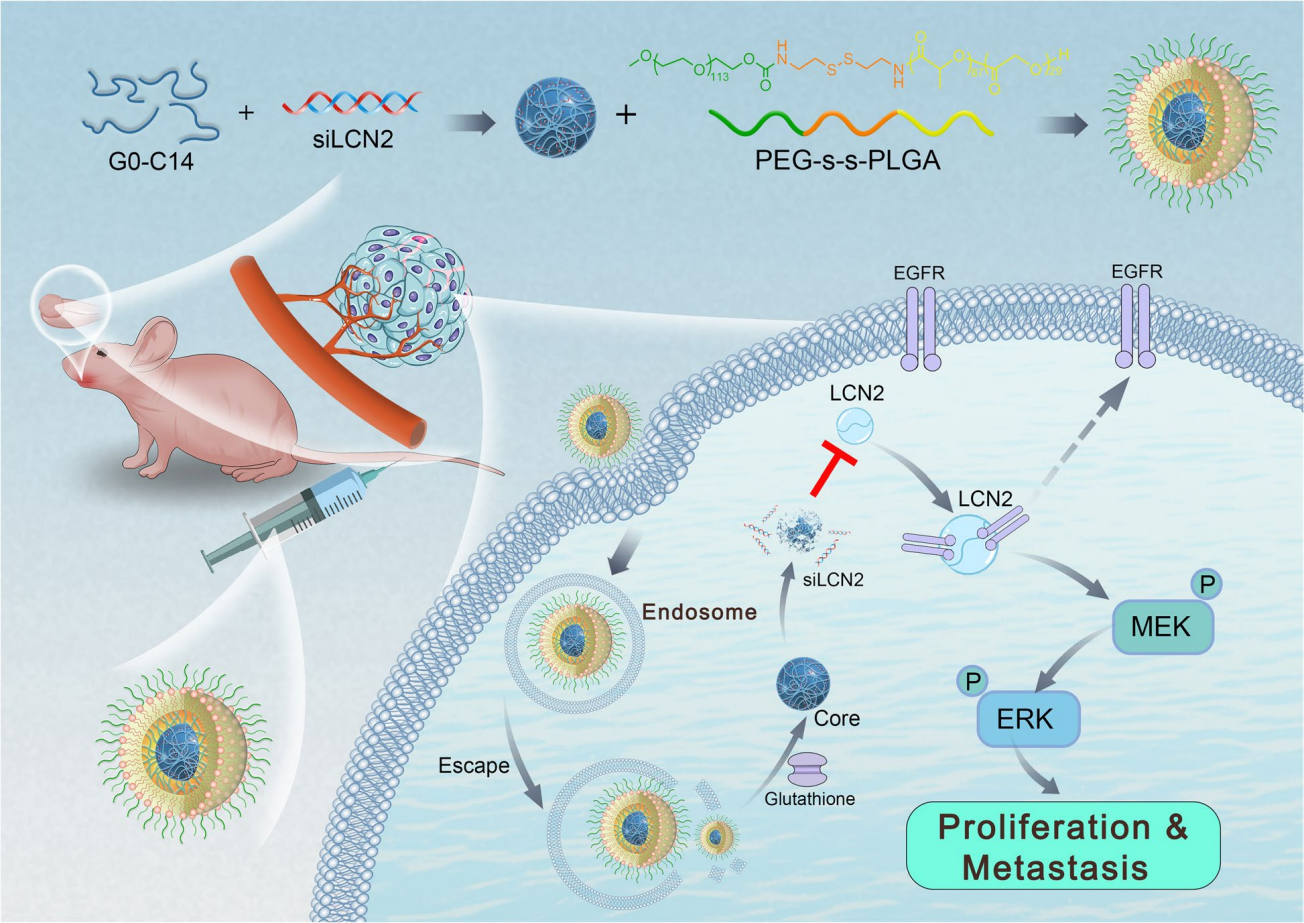
An overview of this study: We used high-throughput sequencing to identify the key factor that regulates the metastasis of oral squamous cell carcinoma (OSCC), LCN2, and revealed that LCN2 promotes the metastatic regulation of OSCC through EGFR activation and recycling. We designed and used glutathione-responsive PEG-SS-PLGA nanoparticles (NPs) containing encapsulated siLCN2. The NPs enter tumor tissue through the EPR effect and are taken up by tumor cells. Through lysosomal escape and the intracellular glutathione response, the NPs release siLCN2, which effectively downregulates the protein expression of LCN2 in tumors and inhibits the EGFR/MEK/ERK signaling pathway. Ultimately, the metastatic function of OSCC cells is inhibited

By analyzing the sequencing profiles, we identified lipocalin 2 (LCN2) as a factor involved in OSCC metastasis and EGFR resistance, indicating that it may work as a potential target; thus, we performed in-depth research on LCN2. Moreover, the use of nanotechnology for nucleic acid-based drug delivery has opened new doors in the field of cancer treatment[11–14]. Encapsulation of siRNA into nanoparticles (NPs) not only enhances siRNA pharmacokinetics to increase the siRNA concentration in cancer cells but also directly inhibits target gene expression, thus limiting cancer malignancy. As proof-of-concept, a reduction-responsive nanoparticle (NP) platform was engineered for effective LCN2 siRNA (siLCN2) delivery, and a tongue orthotopic xenograft model and an EGFR-positive patient-derived xenograft (PDX) model were applied to investigate the curative effect of siLCN2 (Scheme 1).

## Methods

### Antibodies

Antibodies specific for DYKDDDDK (FLAG tag) (#14,793), LCN2 (#44,058), p-EGFR (Tyr1173, #4407), ERK (#4696), p-ERK (#4370) and mouse IgG-HRP (#7076) were purchased from Cell Signaling Technology. Antibodies specific for GAPDH (sc-47724) and β-tubulin (sc-166729) and mouse anti-rabbit IgG-HRP (sc-2357) were purchased from Santa Cruz Biotechnology. Antibodies specific for ERK1/2 (YT1625) and p-ERK1/2 (YP0101) were purchased from ImmunoWay. Antibodies specific for LCN2 (26,991–1-AP), EGFR (66,455–1-Ig, 51,071–2-AP), and KI67 (27,309–1-AP) were purchased from Proteintech.

### Reagents

D-Luciferin was purchased from AAT Bioquest (12,511) and prepared as a 15 mg/mL stock solution in DPBS. The EGFR activator TGF-α (BK0285, Bioworld) was dissolved in anhydrous alcohol (AA) at the appropriate concentration according to the manufacturer's instructions for preservation. Immediately prior to use, the stock solution was diluted to the indicated concentration (10 nM) in culture medium.

Other chemicals were purchased from Sigma. All of the culture media (DMEM for CAL-27 cells and DMEM-F12 for SCC-9 cells) and fetal bovine serum (FBS) were purchased from Biological Industries.

### Plasmids

Human LCN2 and LCN2 short hairpin RNA (shRNA) with the following sequence were purchased from Vigene Biosciences:

GAGCTGACTTCGGAACTAATTCAAGAGATTAGTTCCGAAGTCAGCTCTTTTTT. pMD2. G (#12,259, Addgene) and psPAX2 (#12,260, Addgene) were used as packaging vectors.

To generate overexpression cell lines, the LCN2 sequence was inserted into the pCDH-puro vector between the restriction sites BamHI and NotI. FLAG and SFB tags were added before the N-terminus of LCN2. LCN2-GFP was generated with the lentiviral vector pWPXL (#12,257) between the restriction sites PmeI and BamHI. EGFR-mCherry was generated with pWPXL-LCN2-GFP using HIFI methods by replacing LCN2-GFP.

To generate luciferase-labeled cell lines, the luciferase sequence was inserted into the pCDH- copGFP-neo vector between the restriction sites EcoRI and BamHI.

All of the constructs were confirmed by both DNA sequencing and diagnostic digestion, and the plasmid sequences are shown in the supplemental material.

### Cell culture and transfection

The human OSCC cell lines CAL-27, HN-6 and HEK293t were obtained from the American Type Culture Collection (ATCC). The human erlotinib-resistant OSCC cell lines CAL-27 ER and HN-6 ER were obtained from Prof. Ye.

All OSCC cell lines were routinely cultured in DMEM supplemented with 10% FBS in a 37 °C humidified incubator containing 5% CO2. All the cell lines were validated by short tandem repeat profiling analysis and were free of mycoplasma contamination.

Transient transfection of OSCC cells was performed using Lipofectamine 3000 (Invitrogen) reagent according to the manufacturer’s instructions.

For transient transfection using small interfering RNAs (siRNAs), siRNAs targeting LCN2 and EGFR were synthesized by IGE Biotechnology (Guangzhou, China). Transfection was performed with Lipofectamine RNAiMAX reagent (Invitrogen, USA) according to the manufacturer's protocol. The siRNA sequences were as follows:

siRNA sequences for LCN2 and EGFR.

**Table:** 

Gene		Forward	Reverse
LCN2	s1	CCUCCGUCCUGUUUAGGAATT	UUCCUAAACAGGACGGAGGTT
	s2	GAGCUGACUUCGGAACUAATT	UUAGUUCCGAAGUCAGCUCTT
EGFR	s1	AGGAAUUAAGAGAAGCAACAU	AUGUUGCUUCUCUUAAUUCCUdTdT
	s2	CUCUGGAGGAAAAGAAAGUTT	ACUUUCUUUUCCUCCAGAGTT

For stable expression, lentiviral plasmids harboring the desired gene were first transfected into 293 T cells together with the packaging plasmids pSPAX2 and pMD2.G at a ratio of 5:3:2. HEK293 cells were placed into a 10-cm plate and cultured as previously described. After reaching 70–80% confluence, the cells were transfected with 6 µg of psPAX2, 3 µg of pMD2.G and 10 µg of transfer vector using Lipofectamine 3000 reagent. Forty-eight hours after transfection, the supernatants of each group were collected and used to infect OSCC cells for another 48 h. Puromycin-tolerant or GFP-labeled OSCC cells were picked. Subsequent western blotting and PCR were applied to confirm correct expression in the stable cell lines.

### OSCC sample collection and patient follow-up

For this study, patients who presented at the Department of Oral and Maxillofacial Surgery, Sun Yat-sen Memorial Hospital between 2016 and 2018 for the treatment of OSCC were recruited. The inclusion criteria included a pathological diagnosis of OSCC and willingness to participate in the subsequent follow-up. Patients were excluded as study subjects if they had been diagnosed with multiple cancers or other severe diseases. OSCC patient characteristics, including age, sex, tumor differentiation, lymphatic metastasis and clinical stage, were collected. All patients had a referral at least every season. In addition, tumor samples and adjacent noncancerous (ANC) samples were collected. The ANC tissues were at least 2 cm from the tumor lesion, representing the resection border, and were pathologically confirmed as noncancerous tissues.

### Sample collection and RNA sequencing

OSCC cells with ER and wild type cells were collected and digested using TRIzol (Thermo Fisher Scientific). Specimens were also collected from 2 patients with OSCC and lymph node metastasis and 2 patients with OSCC without lymph node metastasis (basic patient information, Table S1). After freezing with liquid nitrogen, the tissue was homogenized with a tissue grinder, and the tumor tissue was dissolved and digested with TRIzol. RNA integrity was assessed using the RNA Nano 6000 Assay Kit and the Bioanalyzer 2100 system.

Ribosomal RNA was depleted from the total RNA using the rRNA Removal Kit following the manufacturer’s instructions. The RNA was then fragmented into 250 ~ 300-bp fragments, and first-strand cDNA was reverse transcribed using fragmented RNA and dNTPs. The RNA was degraded using RNase H, and second-strand cDNA was synthesized using DNA polymerase I and dNTPs. Sequencing adaptors were ligated to the cDNA. Then, PCR was performed with Phusion high-fidelity DNA polymerase, universal PCR primers and Index (X) primer. The libraries were sequenced with the Illumina NovaSeq platform and subjected to bioinformatic analysis by IGE Biotechnology (Guangzhou, China). We selected HISAT as the mapping tool because HISAT can generate a database of splice junctions based on the gene model annotation file and thus provides a better mapping result than other nonsplice mapping tools. FeatureCounts v1.6.0 was used to count the read numbers mapped to each gene. Then, the FPKM of each gene was calculated based on the length of the gene and read counts mapped to the gene. Prior to differential gene expression analysis, for each sequenced library, the read counts were adjusted by the edgeR program package through one scaling normalized factor. Differential expression analysis of the two conditions was performed using the edgeR R package (3.18.1). A *P* value of 0.05 and absolute fold change of 2 were set as the threshold criteria for significant differential expression. The differentially expressed genes were subjected to Gene On analysis.

### Immunohistochemistry (IHC)

Immunohistochemical staining was performed according to standard protocols. Tumor tissue slides were deparaffinized using xylene and rehydrated with gradient ethanol (100, 95, 90, 80 and 75%) for 5 min. After deparaffinization, antigen retrieval was conducted using 10 mM sodium citrate buffer (pH of 8.0) in a pressure cooker at full power for 10 min. Briefly, the tissue sections were sequentially blocked with 3% H2O2 and normal serum and then incubated with primary antibodies at 4 °C overnight. The tissue sections were incubated with a biotinylated secondary antibody and conjugated with a streptavidin-HRP complex (ready-to-use SP kit; Zhongshan Co., Beijing, China). Finally, the slides were visualized with 3–3'-diaminobenzidine (DAB, CW0125, CW Bio), counterstained with hematoxylin and mounted. The samples were rinsed with phosphate-buffered saline (PBS) between each step.

### Evaluation of IHC staining

IHC tissue staining was evaluated as previously described by 2 pathologists who assessed the number of positive cells and the staining intensity. The positive results were judged based on semiquantitative points. The staining intensity scores were 0 (negative), 1 (weak), 2 (medium) and 3 (strong). The percentage of positive cells was scored as 0 (0%), 1 (1–25%), 2 (26–50%) and 3 (> 50%). The staining intensity score and the proportional score were added to obtain the total score. A total score ≥ 3 was considered to represent high expression. A total score < 3 was considered to represent low expression.

### Western blotting, immunoprecipitation and mass spectrometry

For protein extraction, the cells were washed twice with cool PBS, harvested by scraping and then lysed in lysis buffer (Beyotime, China). Following centrifugation, the supernatant was collected, and the protein concentration was determined using the BCA Protein Assay Kit (PierceTM, USA).

For western blotting, cell lysates were electrophoretically separated on an SDS–PAGE gel using a standard protocol. The proteins were then transferred to polyvinylidene fluoride (PVDF) membranes (IPVH00010; Millipore, USA). The membranes were blocked with 5% nonfat milk in Tris-buffered saline containing 0.1% Tween-20 (TBST) for 1 h at room temperature. The blots were then incubated with the antibodies mentioned above at 4 °C overnight, washed in TBST and probed with secondary antibody. Western blot analysis was performed using the statistical grayscale values from the blots.

For immunoprecipitation, the supernatants were first incubated with S-protein agarose beads (#69,704, Millipore, for SFB-LCN2) overnight at 4 °C, and the precipitates were washed three times with NETN buffer. To detect endogenous interactions, the clarified supernatants were incubated with the antibodies mentioned above for two hours and then with magnetic beads (Pierce™ Protein A/G Magnetic Beads, # 88,802, Thermo Fisher overnight). After being washed three times with NETN buffer, the samples were collected and analyzed by western blotting.

Proteins immunoprecipitated with anti-LCN2 antibody were digested, and the peptides were analyzed by mass spectrometry.

### RNA extraction and real-time quantitative RT–PCR

Total RNA was extracted using TRIzol reagent (Takara, Japan) according to the manufacturer's instructions and then reverse transcribed into cDNA using PrimeScript™ RT Master Mix (Takara, Japan) on an ABI 9700 Real-Time PCR system (ABI, USA). The newly synthesized cDNA was then used as a template for detection of the desired gene.

Specifically, 1 μL of cDNA was mixed with TB Green® Premix Ex Taq™ II (Takara, Japan) in a 20-μl reaction. All of the reactions were run in triplicate using the primers described above. The reaction conditions were as follows: 94 °C for 2 min and 40 cycles of 94 °C for 20 s, 58 °C for 20 s and 72 °C for 20 s. The relative expression of mRNA was detected using the Roche LightCycler 480 II Real-time PCR machine (Roche, USA). The primer sequences were as follows:

Primer sequences for PCR.

**Table:** 

Gene	Forward	Reverse
LCN2	CCCGCAAAAGATGTATGCCA	TCTTAATGTTGCCCAGCGTG
EGFR	CTCCGAGTTGGACGATGAGG	TCATGCCTGCACTGTTCATTC
PRAL	ACCACCGGCATTTCTCCTAAG	AGTCTTTGGGCAGGGCTCAT
TNFSF18	AGCTAGTTCACCAGCACACC	GTAACCTCTGCTTGCCCTGA
GAPDH	GAGTCAACGGATTTGGTCGT	GACAAGCTTCCCGTTCTCAG

### Cell proliferation assay

At 24 h after transfection, the cells were collected, and 2000 cells/well were plated into 96-well plates. The numbers of cells at 24, 48 and 72 h were determined using the MTS Assay Kit (#G3580, Promega, USA). The medium was removed from each well, and 100 μl of 10% MTS in DMEM was added. The plates were incubated for an additional 2 h, and the absorbance at 492 nm was measured using a microplate reader (Multiskan MK3, China). The data are presented as the original OD values.

### Migration and invasion assays

We used a polycarbonate Transwell plate for the migration assay. Specifically, 5 × 104 cells in DMEM containing 1% FBS were seeded into the upper chamber of a Transwell plate (8-µm pore size; Corning, USA). The lower chamber contained DMEM with 15% FBS as a chemoattractant. After the plates were incubated for 24 h in a 37 °C incubator, the medium was removed, and cells on the surface of the upper chamber that did not pass through the membrane were removed with a cotton swab. Cells on the surface of the lower chamber were fixed in paraformaldehyde at room temperature for 20 min and then stained with 0.1% crystal violet for 20 min. Random fields (10 × magnification) were selected, and the cells were counted and photographed.

For the invasion assay, the Transwell plate was precoated with Matrigel (BD, Bedford, MA, USA). The plates were incubated for 48 h before fixation.

### Wound healing assay

Cells were seeded at a density of 6 × 105 into 6-well tissue culture plates. After 24 h of growth, they were transfected with siRNA for another 24 h when they reached approximately 70–80% confluency. For stably transfected cell lines, 8 × 105 cells were seeded into the well and incubated overnight. The monolayer was scraped at the center of the well using a fresh 200-μl pipette tip perpendicular to the plate and scratched in a straight line in one direction. The cells were then washed twice with PBS to remove nonadherent cells, and serum-free medium was added to the plates. The cells were incubated for 48 h, and a photo of the monolayer at the scratch site was taken under a microscope every 12 h (10 × magnification).

### Immunofluorescence staining

Cells were seeded on glass dishes (#D35-20–0-TOP, Cellvis) overnight. The cells were treated with TGF-α for an additional 2 h. At the time of analysis, the cells were washed with PBS, fixed with 4% paraformaldehyde for 20 min, permeabilised with a solution containing 0.1% Triton X-100 in PBS for 10 min and blocked with 1% BSA in PBS for 1 h at room temperature. Primary antibodies (EGFR and LCN2) were then incubated overnight at 4 °C. The slides were washed and secondary antibodies were applied for 1 h in PBS at a final dilution of 1:1000. After washing, the cell nuclei were labelled with DAPI.

### Confocal imaging

To examine LCN2 and EGFR colocalization, OSCC cells were plated on glass-bottomed dishes (#D35-20–0-TOP, Cellvis). The cells were then transfected with the indicated plasmids by using Lipofectamine 3000 (Invitrogen). After transfection for 24 h, the samples were observed and captured on a Zeiss microscope.

For time-lapse imaging of NP uptake by OSCC cells, GFP-endo14 labeled CAL-27 cells at approximately 30–40% confluency were seeded on glass-bottomed dishes for 16 h. After the medium was replaced with 2 mL of fresh medium, glutathione-responsive NPs (G-NPs) loaded with Cy5-labeled siLCN2 were added at an siRNA concentration of 50 nM, and the cells were incubated for 8 h. Images were captured for at least 8 h.

The captured images were analyzed by the Zen Blue microscope imaging software (Zeiss) or ImageJ program. Dozens of cells were selected to define regions of interest (ROIs) for analysis.

### Preparation of G-NPs

The amphiphilic polymer mPEG-SS-PLGA was synthesized according to our previous study and then dissolved in N,N′-dimethylformamide (DMF) to form a homogenous solution with a concentration of 20 mg/mL. Subsequently, a mixture of 1 nmol siLCN2 (0.1 nmol/µL aqueous solution) and 50 µL G0C14 (5 mg/mL in DMF) was prepared and then mixed with 200 µL mPEG-SS-PLGA solution. Under vigorous stirring (1000 rpm), the mixture was added dropwise to 5 mL of deionized water. The NP dispersion was transferred to an ultrafiltration device (MWCO 100 kDa, Millipore) and centrifuged to remove the organic solvent and free compounds. After washing with ultrapure water twice, the obtained G-NPs were dispersed in 1 mL of ultrapure water. The size and zeta potential were determined by dynamic light scattering (DLS, Malvern Zetasizer). The morphology of the saporin-loaded NPs was visualized on a Tecnai G2 Spirit BioTwin transmission electron microscope (TEM). Before observation, the samples were stained with 1% uranyl acetate and dried under air.

To determine the siRNA encapsulation efficiency (EE%), Cy5-labeled siLCN2 was encapsulated into the G-NPs according to the method described above. Subsequently, 5 μL of the NP solution was mixed with 20-fold dimethylsulfoxide (DMSO). The standard was prepared by mixing 5 μL of naked Cy5-siLCN2 (1 nmol/mL) with 20-fold DMSO. The fluorescence intensity was measured using a multimode microplate reader (TECAN SPARK 10 M), and the vector encapsulation efficiency was calculated as EE % = (FINP/FIStandard) × 100. NPs with an EE% greater than 80% were adopted for further experiments.

### In vitro siRNA release

G-NPs loaded with Cy5-siLCN2 were prepared as described above. Subsequently, the G-NPs were dispersed in 1 mL of PBS (pH 7.4) and then transferred to a Float-a-lyzer G2 dialysis device (MWCO 100 kDa, Spectrum) that was immersed in PBS buffer (pH 7.4 with/without 20 nM GSH) at room temperature. At a predetermined interval, 5 µL of the G-NP solution was withdrawn and mixed with 20-fold DMSO. The fluorescence intensity of Cy5 was determined using a microplate reader as described above.

### In vitro gene silencing

OSCC cells were seeded in 12-well plates (50,000 cells per well) and incubated in 1.5 mL of culture medium (pH 7.4) with 10% FBS for 24 h. Thereafter, the medium was replaced with fresh medium, and G-NPs loaded with siLCN2 were added at a siRNA concentration of 50 nM. After 24 h of incubation, the cells were washed with PBS buffer (pH 7.4) and allowed to incubate in fresh medium (pH 7.4) for another 48 h. After removing the medium and subsequently washing with PBS buffer (pH 7.4) three times, the cells were collected for q-PCR and immunoblot analysis of LCN2 expression.

### Pharmacokinetics study

Healthy female nude mice were randomly divided into two groups (*n* = 5) and given an intravenous injection of either naked Cy5-siRNA or Cy5-siRNA-loaded G-NPs at a 1-nmol siRNA dose per mouse. At predetermined time intervals, orbital vein blood (20 µL) was withdrawn using a tube containing heparin, and the wound was pressed for several seconds to stop the bleeding. The fluorescence intensity of Cy5-labeled siRNA in the blood was determined by a microplate reader.

### Biodistribution

Tumor-bearing nude mice were randomly divided into two groups (*n* = 5) and given an intravenous injection of either naked Cy5-siRNA or Cy5-siRNA-loaded G-NPs at a 1-nmol siRNA dose per mouse. Twenty-four hours after the injection, the mice were imaged using the IVIS system (Cri Inc.). The organs and tumors were then harvested and imaged. To quantify the accumulation of G-NPs in tumors and organs, the tissues were homogenized, and the fluorescence intensity of Cy5-siLCN2 in each organ was examined by a microplate reader. The Cy5 intensity is presented as (total intensity)/organ weight.

### OSCC tongue orthotopic xenograft modeling and NP application

To explore the effects of LCN2 on OSCC tumors in vivo, CAL-27 luciferase-labeled cells and LCN2-stable cell lines were used to prepare tumor xenografts. OSCC cells (1.0 × 106) were implanted into the left tongue edge of BALB/c nude mice (female, 6 weeks, 18–20 g). The tumors were measured by the IVIS system every week.

When the tumor was recognized by the IVIS system and displayed luciferase luminescence, NPs were prepared for treatment. The NPs were administered by tail vein injection every 2 days for a total of 3 times, and 10 nM siLCN2 was administered each time. Subsequently, we performed luciferase luminescence detection of the mouse tongue tumors every week and measured the weight of the mice at the same time.

The tumors, cervical lymph gland, viscera, and peripheral blood were harvested for further detection after 3 weeks.

### PDX model and NP application

To generate a PDX model of OSCC, a specific OSCC patient was selected. He was treated with cetuximab first and underwent surgical resection due to tumor recurrence due to drug resistance. We also performed histological examination and found that the OSCC sample displayed strong EGFR positivity (Fig. S7). The tumor tissues were cut into small pieces and subcutaneously transplanted into the right upper back of NSG (NOD/SCID/IL2Rγ null) mice (female, 5 weeks, 16–18 g). When the tumor volume reached approximately 60 mm3, NPs encapsulating siLCN2 were prepared for treatment. The NPs were administered by tail vein injection every 2 days for a total of 3 times. The mice were sacrificed 20 days after treatment began. Then, the tumors were harvested for IHC detection. The organs and peripheral blood were harvested for toxicity detection.

### Statistical analyses

All statistical analyses were conducted using SPSS 19.0 statistical software. Kruskal–Wallis analysis was used to examine the relationships between clinicopathological characteristics and protein expression. The survival curves were plotted using the Kaplan–Meier method and compared with the log-rank test. Fisher’s exact test was used to examine the relationships between LCN2 expression and xenograft features. Student's t test was used to compare the PCR results, tumor xenograft results, and differences in cell function (proliferation, migration, invasion, etc.) between the different groups. Unless otherwise noted, quantitative data are expressed as the mean and standard error of the mean (S.E.M.). Statistical significance was determined with a paired Student’s t test (∗ *P* < 0.05; ∗  ∗ *P* < 0.01; ∗  ∗  ∗ *P* < 0.001, compared with the control).

## Results

### EGFR-resistant OSCC cell lines displayed high metastatic abilities

**Fig. 1:**
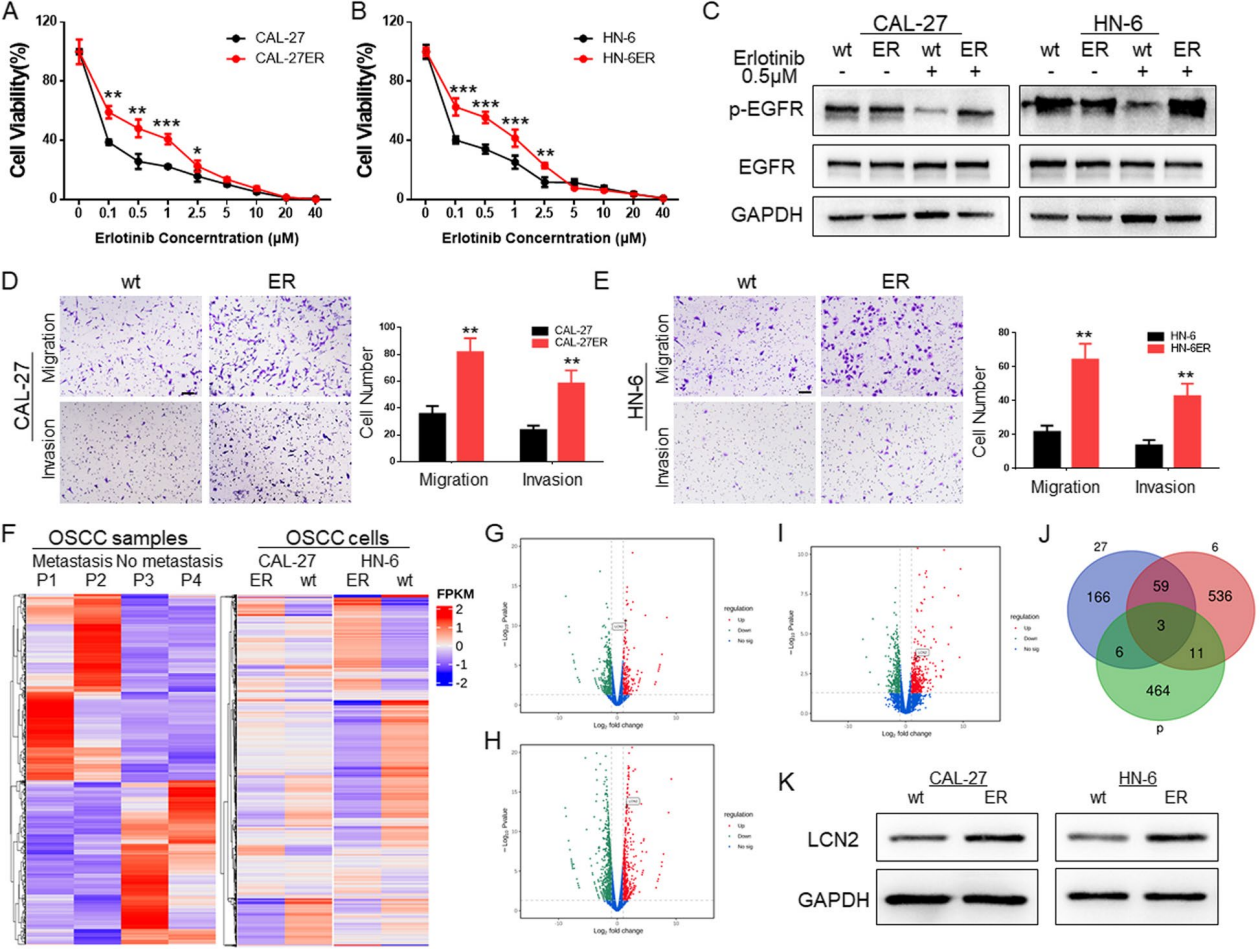
**A** The effect of erlotinib at gradient concentrations on CAL-27 and CAL-27ER cells. The viability of CAL-27ER cells was higher at the same concentration, and there was a significant difference between 0.1 μM and 2.5 μM. **B** The effect of gradient concentrations of erlotinib on HN-6 and HN-6ER cells. The cell viability of HN-6ER cells at the same concentration was higher, and there was a significant difference between 0.1 μM and 2.5 μM. **C** CAL-27 and HN-6 EGFR-resistant strains showed no significant change in p-EGFR expression under the EGFR inhibitor (erlotinib) at a concentration of 0.5 μM, while that of the wild-type strain significantly decreased. **D** The migration and invasion abilities of the CAL-27 ER cell line were stronger than those of the wild type, and the number of cells passing through the Transwell chamber at the same time was greater than that of the wild type. (Scale bar: 100 μm). **E** The migration and invasion abilities of the HN-6 ER cell line were stronger than those of the wild type, and the number of cells passing through the Transwell chamber at the same time was greater than that of the wild type. (Scale bar: 100 μm). **F** OSCC patients with or without cervical lymph node metastasis and OSCC cell line sequencing and heatmap analysis. **G** Volcano plot of OSCC patient specimen sequencing data. LCN2 was identified among the highly expressed genes in the metastatic group. **H** Volcano plot of data from CAL-27 cells and their ER cells. LCN2 was identified among the highly expressed genes in ER cells. **I** Volcano plot of data from HN-6 and its ER cells. LCN2 was identified among the highly expressed genes in ER cells. **J** Venn diagram of genes that were upregulated before and after transfer obtained by sequencing OSCC patients and OSCC cell lines; a total of 3 genes were upregulated. **K** Western blotting of the sequencing results confirmed that LCN2 expression was significantly increased in highly metastatic OSCC cells

We treated CAL-27 and HN-6 cells and their EGFR inhibitor-resistant strains (ERs) with an EGFR inhibitor (erlotinib). From the drug resistance detection results, it was found that the cell viability of ER-resistant strains was higher than that of wild-type cells at concentrations from 0.1 μM to 2.5 μM (Fig. 1A and B). After 12 h of treatment with erlotinib at a concentration of 0.5 μM, the protein was extracted for detection. The expression of p-EGFR and EGFR in the wild-type cell line was significantly lower than that in the ER cell lines (Fig. 1C). Subsequently, we compared the migration and invasion abilities of ER cell lines with those of wild-type cell lines. The results also showed that the metastatic abilities of ER drug-resistant strains were significantly enhanced (Fig. 1D and E).

### LCN2 is involved in lymph node metastasis regulation and EGFR resistance in OSCC

The regulation of EGFR expression is related not only to the drug resistance of tumor cells but also to the regulation of tumor lymph node metastasis. Due to the emergence of EGFR inhibitor resistance, to find more effective and new tumor regulation sites, we specifically selected specimens from OSCC patients with or without lymph node metastasis under the same T2 stage classification in this study (Table S1) and compared their mRNA expression profiles. In addition, the CAL-27, HN-6 and ER-resistant strains were sequenced together. Genes that were significantly differentially expressed in metastatic tissues and ER cell lines were examined (Fig. 1F).

According to the sequencing results, we selected the genes that were highly expressed in metastatic tissues and drug-resistant cell lines (Fig. 1G-J), performed Venn diagram analysis, and found that 3 genes statistically participated in both processes (Table S2).

To further determine which of the three is the ideal candidate, we performed qPCR verification at the cell level. LCN2 expression was the most drastically and consistently altered, while TNFSF18 displayed very low and variable expression, and PRAL expression trends differed at the sequence and mRNA expression levels(Figs. 1K and S1). Moreover, LCN2 has been reported to be involved in the drug resistance process and regulation of EGFR activation [15, 16]. Hence, we selected it as the potential target in this research. Then, we verified that LCN2 expression is elevated in the wild type and ER cell of CAL-27 and HN-6.

### LCN2 expression positively correlated with EGFR expression and poor prognosis in OSCC

**Fig. 2:**
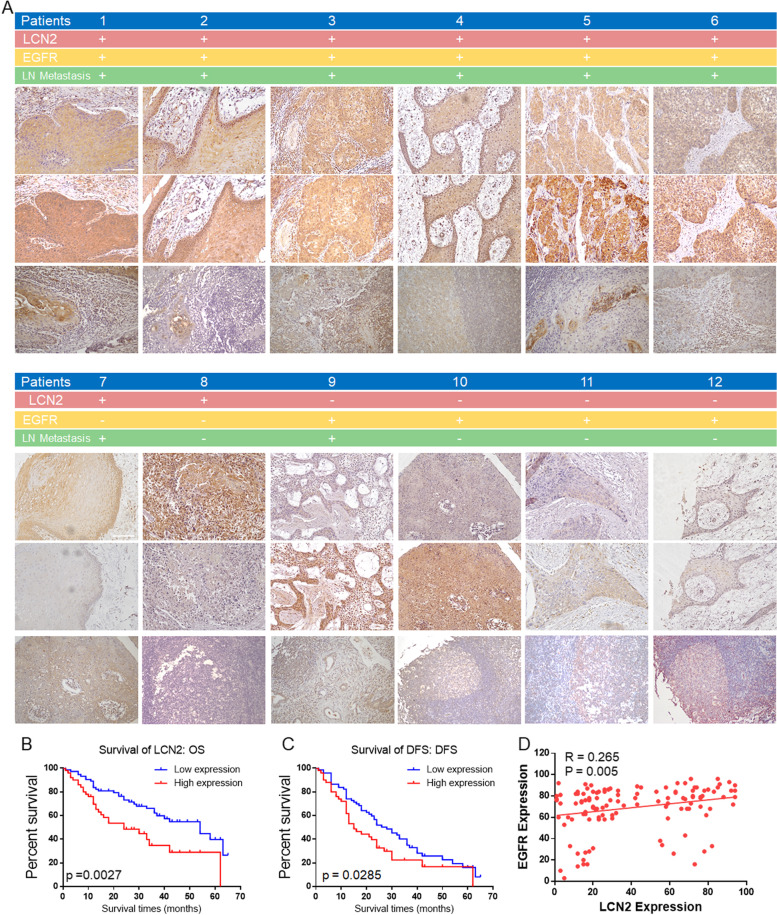
**A** Immunohistochemical detection of LCN2 and EGFR expression in OSCC patient specimens. LCN2 expression positively correlated with EGFR expression. High LCN2 and EGFR expression was accompanied by cervical lymph node metastasis. (Scale bar: 100 μm). **B** Higher levels of LCN2 in OSCC were associated with poor overall survival (OS) in OSCC patients (*n* = 124, *p* = 0.0027). **C** Higher levels of LCN2 in OSCC were associated with poor disease-free survival (DFS) in OSCC patients (*n* = 124, *p* = 0.0285). **D** The positive correlation between LCN2 expression and EGFR expression in OSCC tissues

We need to clarify the relationship between the expression of LCN2 in patients with OSCC and the clinical characteristics of the patients. Hence, 124 OSCC patients who were treated at Sun Yat-sen Memorial Hospital, Sun Yat-sen University from 2016–2018 were enrolled. Their clinical features and follow-up information were collected. Immunohistochemical analysis of LCN2 and EGFR and correlation analysis of the clinical features of the OSCC patients were further performed (Fig. 2A).

The results showed that LCN2 expression was positively correlated with EGFR expression (Fig. 2D). The expression of LCN2 was related to OSCC lymph node metastasis, differentiation and T stage but not age or sex (Table 1). Moreover, high LCN2 expression was correlated with poor overall survival (OS, Fig. 2B) and disease-free survival (DFS, Fig. 2C) in OSCC patients. Studies have found that LCN2 is involved in the regulation of various tumor functions and can be used as an independent predictor of tumor patient prognosis [17–19]. Therefore, LCN2 is not only related to EGFR expression but also has the potential to be an important target for OSCC regulation, making it worthy of further investigation.

**Table 1:** Association of LCN2 expression with the features of OSCC patients

Clinical characteristics	LCN2 expression		*P* value
	High expression(%)	Low expression(%)	
Age			
≤ 40	13(39.39)	20(60.61)	0.7135
40–50	10(47.62)	11(52.38)	
50–60	11(45.83)	13(54.17)	
> 60	16(34.78)	30(65.22)	
Gender			
Male	32 (41.56)	45(58.44)	0.7195
Female	18 (38.30)	29(61.70)	
Differentiation			
High	17 (29.31)	41(70.69)	0.0179
Mieddle	19 (43.18)	25(56.82)	
Low	14 (63.64)	8(36.36)	
Lymphatic metastasis			
No	19 (29.23)	46(70.77)	0.008
Yes	31 (52.54)	28(47.46)	
Clinical T stages			
1	6 (20.00)	24(80)	0.015
2	13 (35.14)	24(64.86)	
3	15 (51.72)	14(48.28)	
4	16(57.14)	12(42.86)	

LCN high expression:50 cases(40.32%); low expression:74 cases(59.68%)

### LCN2 expression regulates the metastatic functions of OSCC

**Fig. 3:**
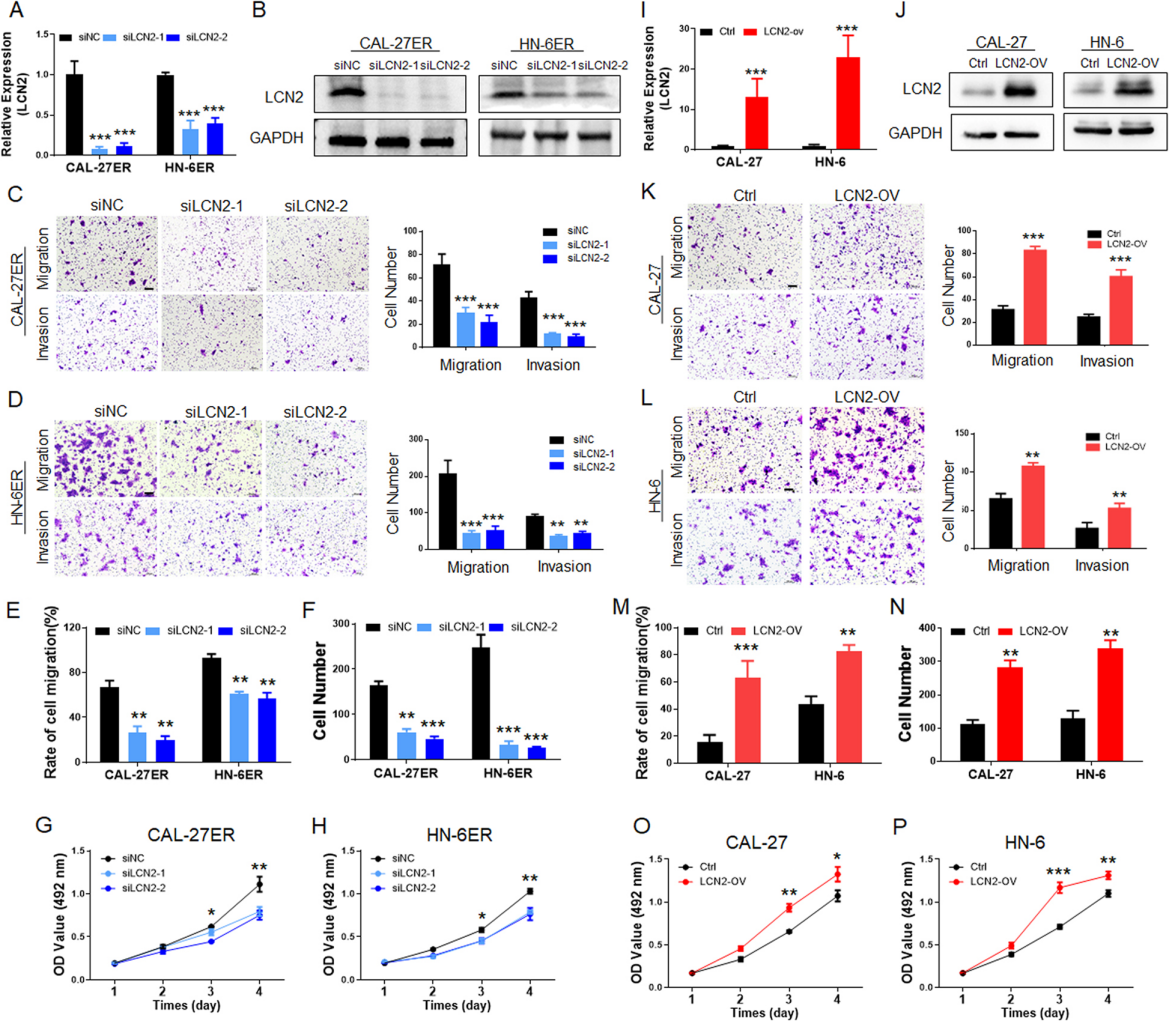
**A** PCR detection confirmed that LCN2 was successfully inhibited in the EGFR-resistant OSCC cell lines. **B** Western blotting confirmed that LCN2 was successfully inhibited in CAL-27ER and HN-6ER cells. **C** and **D** After the expression of LCN2 was downregulated, the migration and invasion of CAL-27ER cells were significantly inhibited, and the cells that passed through the upper chamber of the Transwell were significantly reduced. (Scale bar: 100 μm). **E** The scratch test showed that LCN2 was downregulated, the migration function of CAL-27ER cells was significantly downregulated, and the scratch healing speed (recovery rate) was decreased. **F** The cell colony formation test showed that after inhibiting LCN2 in ER-resistant cells, the colony formation of OSCC cells decreased significantly. **G** and **H** Inhibiting the expression of LCN2 significantly decreased the proliferation ability of OSCC cells, and the CCK-8 results were lower than those of the control group. **I** PCR detection confirmed that LCN2 was successfully overexpressed in wild-type CAL-27 and HN-6 cells. **J** Western blotting showed that LCN2 was successfully overexpressed in wild-type CAL-27 and HN-6 cells. **K** and **L** After overexpression of LCN2, the migration and invasion of CAL-27 cells were significantly upregulated, and the number of cells that passed through the upper chamber of the Transwell was significantly increased. (Scale bar: 100 μm). **M** After the scratch experiment confirmed that LCN2 was overexpressed, the migration ability of CAL-27 cells was significantly upregulated, and the scratch healing speed was increased. **N** The cell colony formation assay showed that the colony formation of OSCC cells increased significantly when LCN2 was overexpressed. **O** and **P** Upregulation of LCN2 in OSCC cells significantly increased their proliferation abilities, and the CCK-8 values were higher than those of the control group

To evaluate the potential of LCN2 in OSCC regulation, we examined both metastasis and proliferation. After successfully inhibiting the expression of LCN2 in CAL-27ER and HN-6ER cells by siRNA transfection (Fig. 3A and B), migration and invasion experiments (Fig. 3C and D) designed in Transwell chambers and cell scratch experiments (Figs. 3E and S2A) were performed. It is clear that when the expression of LCN2 was decreased in OSCC cells, the metastatic ability of the cells was significantly suppressed. Subsequently, a cell colony formation assay (Figs. 3F and S2C) and CCK-8 (Fig. 3G and H)-based proliferation assay also clarified the downregulation of OSCC cell proliferation ability after LCN2 expression inhibition.

In contrast, when we constructed LCN2-overexpressing cell lines in CAL-27 and HN-6 cells by lentiviral transfection (Fig. 3I and J), the cell biological functions were significantly enhanced. The number of cells in the LCN2-OV group that passed through the lower chambers of Transwell compartments was significantly higher than that of the control group (Fig. 3K and L). Scratch assays also confirmed that the wound healing efficiency was higher in the LCN2-OV group than in the control group (Figs. 3M and S2B). Cell proliferation assays displayed more colony formation (Figs. 3N and S2D) and higher CCK-8 reads at the same times (Fig. 3O and P).

### LCN2 promotes ERK signal activation and interacts with EGFR

**Fig. 4:**
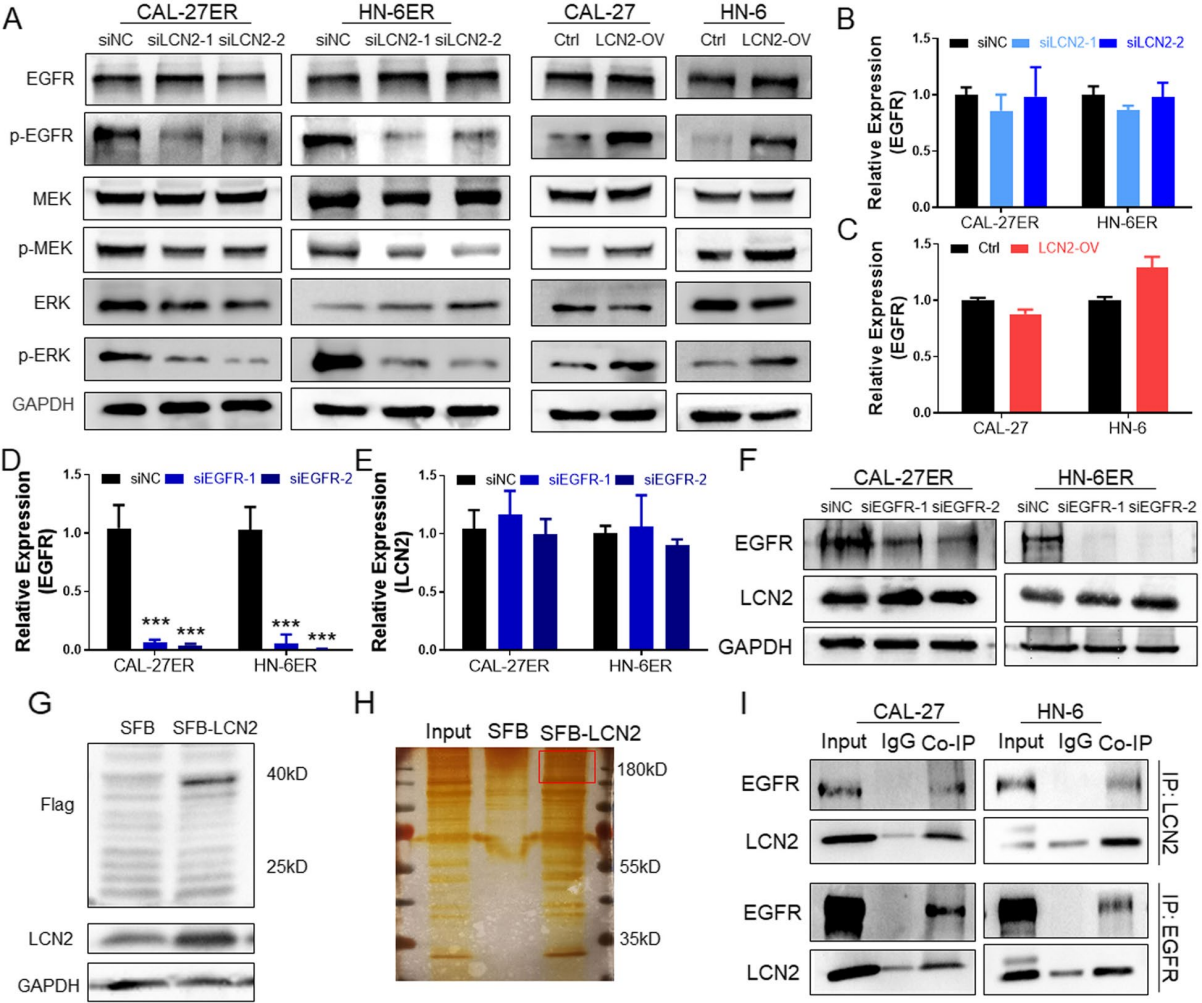
**A** After LCN2 was downregulated in OSCC ER cells, the expression levels of p-EGFR, p-MEK, and p-ERK were also significantly downregulated. In contrast, when LCN2 was upregulated in OSCC cells, the expression levels of p-EGFR, p-MEK, and p-ERK were upregulated. **B** LCN2 downregulation had no significant effect on EGFR mRNA expression. **C** LCN2 upregulation had no significant effect on EGFR mRNA expression. **D** The mRNA expression of EGFR was successfully downregulated by siEGFR in OSCC cell lines. **E** Inhibition of EGFR expression had no significant effect on LCN2 mRNA expression. **F** Western blot detection confirmed that EGFR was successfully inhibited but had no significant effect on LCN2 protein expression. **G** Construction of stably transfected CAL-27 cells labeling LCN2 with the SFB tag and verification by immunoblotting. **H** Protein silver staining results after co-IP. EGFR is indicated in the red frame. **I** Mutual co-IP and immunoblotting detection confirmed the interaction between EGFR and LCN2

To explore the regulatory mechanism of LCN2 in OSCC, we examined the expression of the EGFR/MEK/ERK pathway. EGFR phosphorylation was significantly decreased after LCN2 was inhibited (Fig. 4A) and enhanced after LCN2 was overexpressed (Fig. 4B). The downstream factors MEK and ERK displayed the same trend as LCN2-regulated EGFR. However, the total protein expression of EGFR was not significantly changed by LCN2. Further study showed that the change in LCN2 mRNA expression had no significant effect on EGFR mRNA expression (Fig. 4B and C). Inhibition of EGFR expression also did not affect LCN2 expression (Fig. 4D-F).

Combined with the above experimental results, when the expression of LCN2 increased, the activation of P-EGFR increased as well. However, when the expression of LCN2 and EGFR decreased, the mutual expression regulation between them was not obvious. However, the phosphorylation regulation of the EGFR pathway is significant. Therefore, we infer that the regulation of EGFR expression by LCN2 is a protein‒protein interaction.

We designed an LCN2 overexpression plasmid with the SFB tag and constructed the corresponding stable cell line (SFB-LCN2, Fig. 4G). LCN2 coimmunoprecipitation (co-IP) and mass spectrometry analysis confirmed that the protein that interacts with LCN2 was enriched in the ERK signaling pathway and binds EGFR (Figs. 4H, S3A and B, Supply mass analysis). Subsequently, the interactions between LCN2 and EGFR were confirmed by co-IP and immunoblotting (Fig. 4I).

### LCN2 promotes EGFR recirculation to increase EGFR pathway activation

**Fig. 5:**
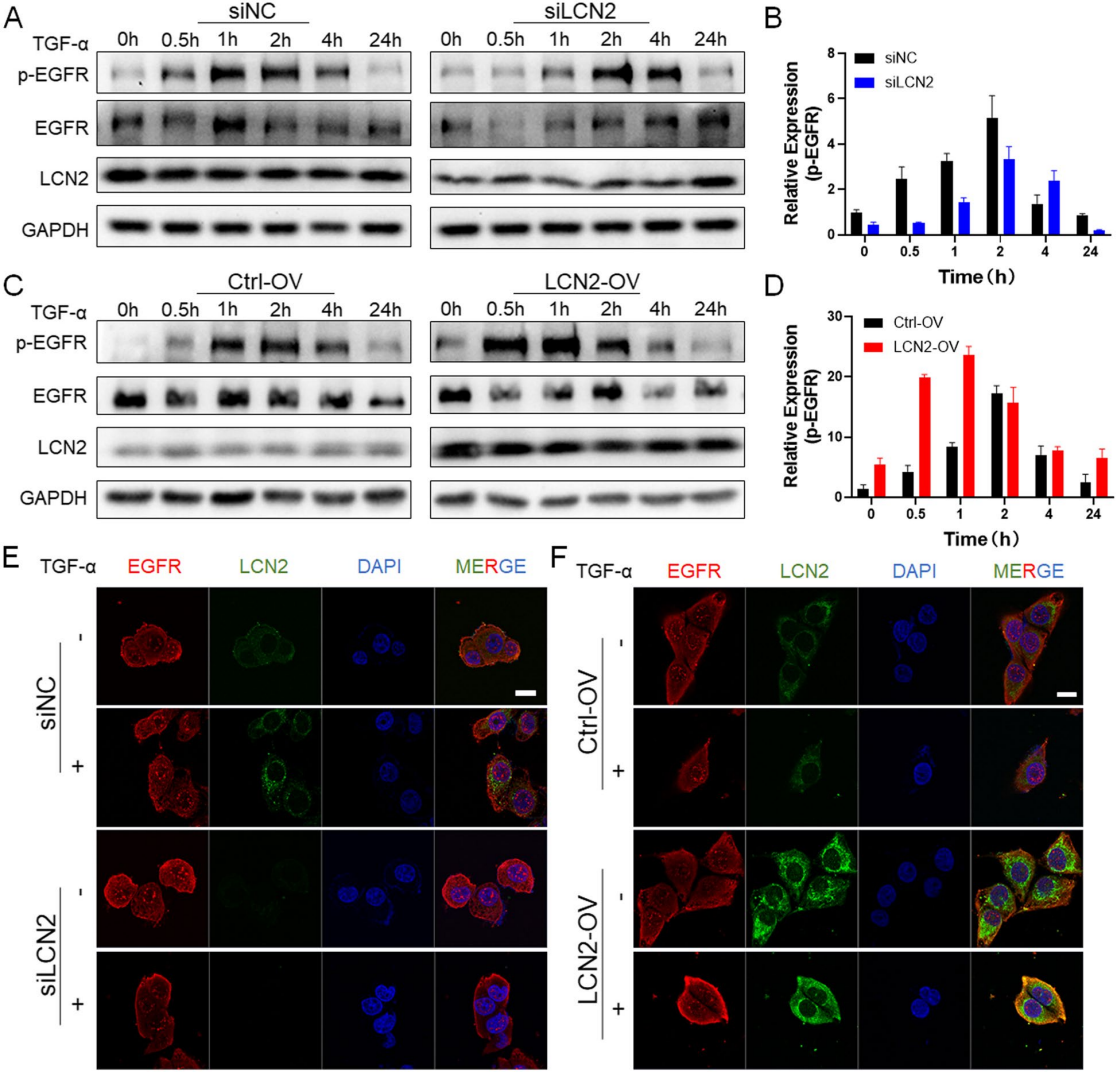
**A** In the LCN2-knockdown group, after treatment with TGF-α, EGFR activation was significantly increased after 2 h and quickly dissipated after 4 h, while the results in the control group were the same as those in the ctrl-OV control group, showing activation after 1 h and dissipation after 4 h. **B** p-EGFR gray value statistics. After inhibiting the expression of LCN2, p-EGFR appeared slowly but dissipated rapidly. **C** In LCN2-overexpressing cells, after TGF-α treatment, p-EGFR was rapidly activated in the LCN2-OV group and gradually dissipated after 4 h, but p-EGFR in the control group cells was only activated after 1 h and dissipated after 4 h. **D** p-EGFR gray value statistics. p-EGFR quickly appeared and slowly dissipated after LCN2 was upregulated. **E** After the expression of LCN2 was inhibited, a small amount of EGFR was transferred from outside of the cell membrane to the inside of the cell after TGF-α stimulation, but the degree of transfer was less than that of the control group, and there was no significant difference. (Scale bar: 20 μm). **F** IF analysis showed that after LCN2 overexpression, EGFR localization rapidly shifted from an extramembranous to an intracellular location after TGF-α treatment, and colocalization with LCN2 increased, while in the control group, EGFR was still mostly located on the cell membrane, and a small amount entered the cells to colocalize with LCN2. (Scale bar: 20 μm)

EGFR is initially distributed on the cell membrane. When it is activated, it enters the cell and activates MEK phosphorylation, initiating downstream ERK signaling, which promotes tumor metastasis. Activation of the EGFR pathway is ligand- or kinase-dependent [20], and EGFR tends to be recycled or degraded in the endosome, respectively [21]. Based on the results, we speculated that the binding of LCN2 to EGFR promotes EGFR recycling. Therefore, TGF-α was used to activate EGFR in OSCC cells. The results showed that in the LCN2-OV group, TGF-α quickly induced the phosphorylation of EGFR to a greater extent than EGFR phosphorylation in the control group (Fig. 5C and D). Intense phosphorylation occurred beginning at 0.5 h and persisted until 4 h. In the control group, phosphorylation occurred from 1 h, and it was significantly decreased at approximately 4 h. EGFR in siLCN2 cells slowly responded to TGF-α, after which phosphorylated EGFR quickly disappeared (Fig. 5A and B).

Subsequently, we observed changes in this process in cells. Hence, we constructed LCN2-GFP and EGFR-mCherry plasmids and transfected them into HEK293T cells. The results showed that EGFR was initially distributed on the cell membrane, while LCN2 was located in the cytoplasm. EGFR and LCN2 displayed weak colocalization. When the cells were stimulated with TGF-α, EGFR quickly entered the cytoplasm (Fig. S4A, Movie 1). TGF-α-activated EGFR can be reprocessed in the endoplasmic reticulum and reattach to the membrane. Additionally, green fluorescence (LCN2) and red fluorescence (EGFR) clearly colocalized and gathered in the endoplasmic reticulum, which tended to aid the recovery and reprocessing of EGFR to complete its recycling.

To further clarify whether LCN2 binds to EGFR and participates in EGFR activation and recycling, immunofluorescence (IF) detection in LCN2-transfected cells was performed. After the application of TGF-α, the colocalization rate of LCN2 and EGFR was greater in the LCN2-OV group than in the control group (Fig. 5F) and lower in the siLCN2 group than in the control group (Fig. 5E). We quantitatively and statistically analyzed the fluorescence intensity of the membrane signal of EGFR. The signal was significantly enhanced by approximately twofold in LCN2-OV cells (Fig. S4B), and 1/2 in siLCN2 cells before and after TNF-α-stimulated.

Therefore, LCN2 stimulates the functions of OSCC by promoting the activation and recycling of EGFR.

### LCN2 expression regulates the progression of OSCC tongue orthotopic xenografts

**Fig. 6:**
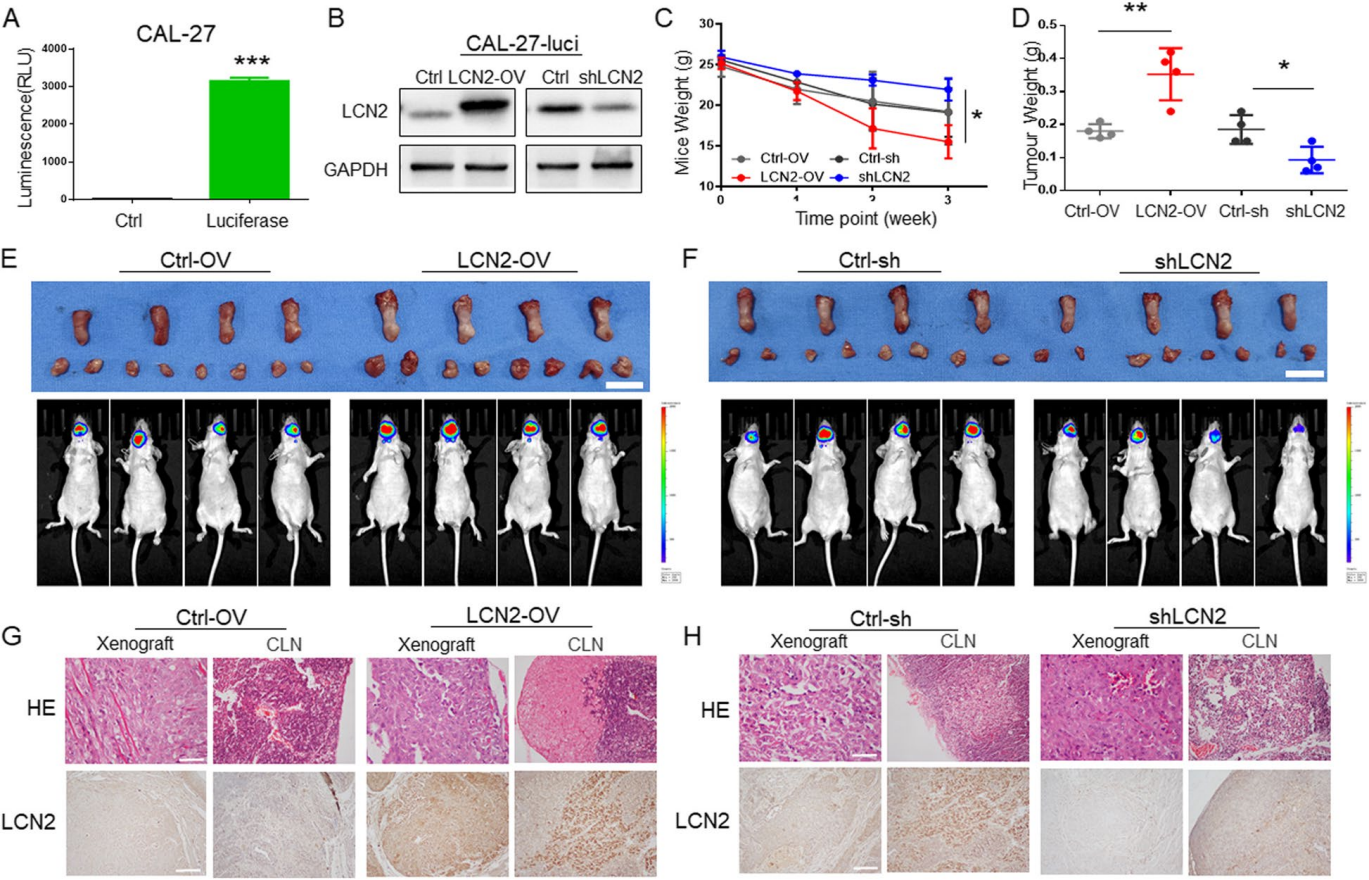
**A** After luciferase-labeled CAL-27 cells were constructed, the successful construction of the cell line was confirmed by luciferase fluorescence detection. **B** LCN2-overexpressing and LCN2-knockdown CAL-27-Luci cells were constructed by lentiviral transfection and confirmed by immunoblotting. **C** After a mouse tongue in situ tumor model was constructed, changes in mouse body weight were detected; the weight loss of the LCN2-OV group was greatest, and the weight loss of the shLCN2 group was lowest. **D** In situ tumors from the mouse tongue were dissected and weighed. The LCN2-OV group had the heaviest tumors, indicating that the tumors were the largest, while the shLCN2 group had the lightest tumors, indicating the smallest tumor volume. **E** After overexpression of LCN2, all mice with in situ tumors of the tongue developed lymph node metastasis (small dots in the submandibular region), while lymph node metastasis occurred in 1 mouse in the control group. (Scale bar: 1 cm). **F** After the expression of LCN2 was inhibited, all of the mice with in situ tumors of the tongue developed lymph node metastasis (small dots in the submandibular region), while lymph node metastasis occurred in 1 mouse in the control group. **G** Representative images of tongue tumors and cervical lymph nodes in the LCN2-ov group. HE staining showed tongue tumors and lymph node metastases in the LCN2-overexpressing group, and overexpression of LCN2 was confirmed by IHC staining. (Scale bar: 100 μm). **H** Representative images of tongue tumors and cervical lymph nodes in mice in the shLCN2 group. HE staining showed tongue tumors and lymph node metastases in the control group, but no metastases were found in the shLCN2 group, as detected by HE staining. The expression of LCN2 was clearly inhibited, as shown by IHC color development. (Scale bar: 100 μm)

To further verify the functions of LCN2 in vivo, a luciferase-labeled CAL-27 (CAL-27-Luci, Fig. 6A) was constructed, and cell lines in which LCN2 was overexpressed (LCN2-OV) or knocked down (shLCN2) were further constructed by lentivirus transfection (Fig. 6B). An OSCC orthotopic xenograft model was established in nude mice by the implantation of CAL-27-Luci cells on the left lateral edge of the tongue. Intravital imaging was performed weekly.

The tumors in the LCN2-OV group displayed cervical lymph node metastasis (Fig. 6E), while those in the shLCN2 group exhibited no lymph node metastasis and tended to display tumor shrinkage (Fig. 6F). HE staining confirmed OSCC lymphatic metastasis results from IVIS images by revealing OSCC cells in the cervical lymph nodes (Fig. 6G and H). The mouse weight and xenograft weight changed in opposite ways: the LCN2-OV group displayed the highest xenograft weight, and their host-mouse weight decreased rapidly (Fig. 6C and D). The opposite trend was observed in the shLCN2 group.

Although there was a significant difference between the LCN2-OV group and shLCN2 group in lymph node metastasis and mouse weight, there was no significant difference between the LCN2-OV and shLCN2 groups and their corresponding control groups. This may be due to the small number of animals, but as a trend study, it has been well documented that inhibiting LCN2 expression successfully suppressed OSCC tumor proliferation and metastasis and could be a potential target for OSCC treatment.

### NPs delivered siLCN2 to inhibit the functions of OSCC cells

**Fig. 7:**
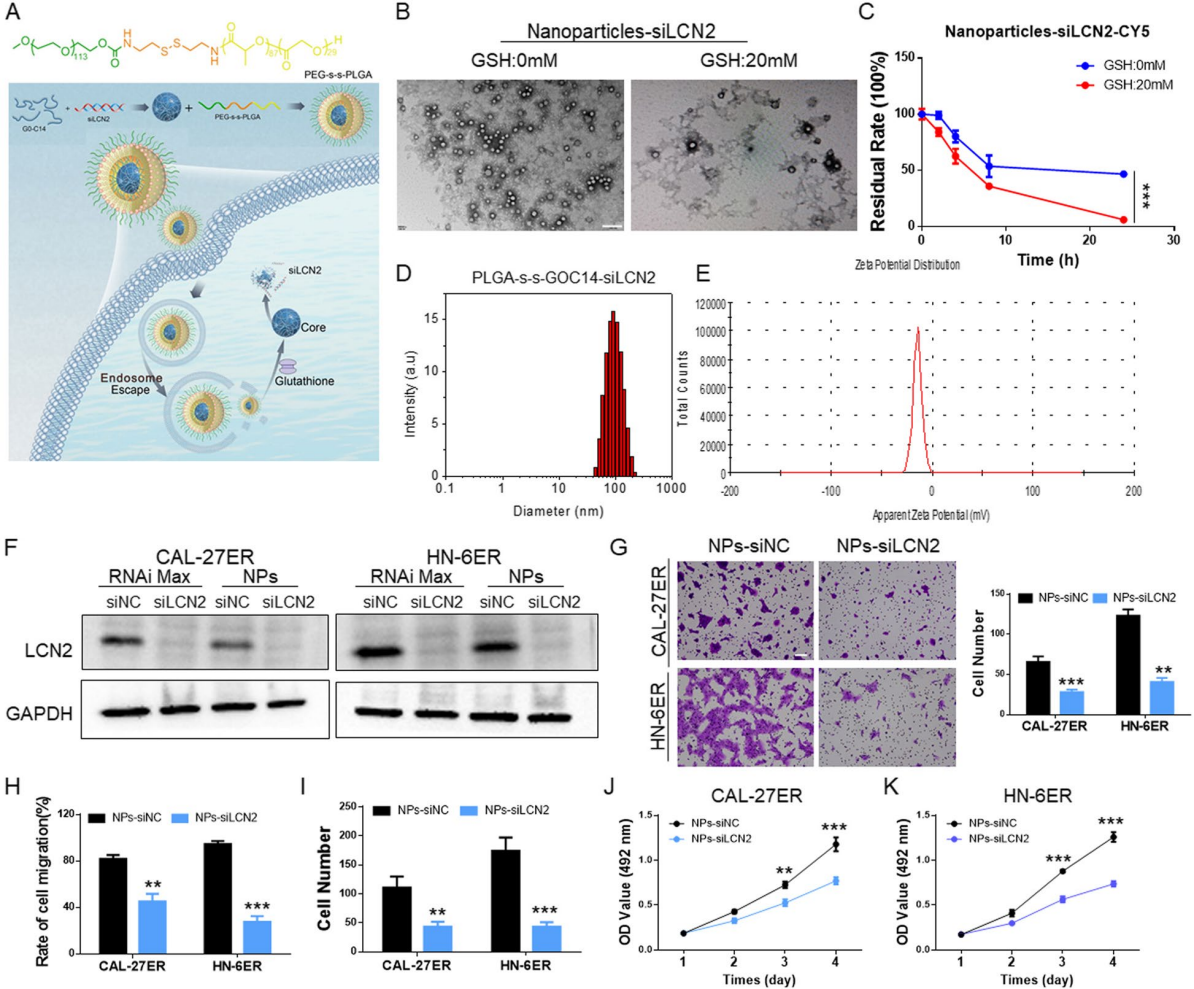
**A** Schematic diagram showing the structure of mPEG-SS-PLGA nanoparticles and their release of siLCN2 in cells through lysosomal escape and the response to glutathione. **B** Electron microscopy images showing the particle size of the nanoparticles and their release in the presence of 20 mM glutathione. (Scale bar: 200 nm). **C** After the nanoparticles encapsulated siLCN2, the release of siLCN2-Cy5 in the presence of glutathione (20 mM) was monitored. At a glutathione concentration of 20 mM, more siLCN2 was released. **D** Nanoparticle size detection; the nanoparticle size was between 80–120 nm. **E** Nanoparticle potential detection; the potential was approximately -20 mV. **F** OSCC cells were transfected with RNAiMAX transfection reagent and nanoparticle transfection reagent. Western blotting showed that both transfection methods significantly inhibited the expression of LCN2. **G** After LCN2 expression was downregulated in OSCC cells, the migratory abilities of the cells were significantly reduced. (Scale bar: 100 μm). **H** The scratch healing assay showed that after LCN2 expression was downregulated in CAL-27ER and HN-6ER cells by NPs, cellular migration was decreased. **I** The cell colony formation test showed that the colony formation of OSCC cells decreased significantly in the NPs-siLCN2 groups. **J** and **K** Inhibition of LCN2 in OSCC cells by NPs significantly decreased their proliferation abilities, and the CCK-8 results were lower than those of the control group

The aforementioned in vivo and in vitro experiments demonstrated that LCN2 regulates the functions of OSCC via EGFR signaling. As a preclinical experiment, we designed NPs, PEG-SS-PLGA, which is responsive to glutathione in the tumor microenvironment, for the encapsulation and delivery of siRNA-LCN2 (Fig. 7A). After synthesis of the NPs, DLS was applied to detect the basic characteristics. The NP size was within 120 nm (Fig. 7D), and its potential was -14 mV (Fig. 7E). Then, electron microscopy was applied for observation. In the presence of glutathione, the NPs quickly degraded and released the encapsulated siRNA (Fig. 7B). This observation was confirmed by in vitro siRNA release (Fig. 7C), which showed that the residual rates of siLCN2 were significantly lower in 20 nM GSH than in PBS. Therefore, the NPS that we designed and synthesized matched the criteria of a nanoencapsulated material.

The delivery of siLCN2 to OSCC cells was first verified. We loaded the NPs with Cy5-labeled siLCN2 and incubated them with OSCC cells. Within 8 h of incubation at 37 °C, the Cy5 fluorescence signal quickly moved into the cell, indicating siLCN2 endocytosis from outside the cell membrane into the cell, its concentration in the endosome, and then its release (Fig. S5A). NP-encapsulated siLCN2 was transfected into OSCC cells for 48 h, and the protein was extracted to verify the successful downregulation of LCN2 expression (Fig. 7F) and the inhibition of OSCC cell migration (Figs. 7G and H, S5B) and proliferation (Figs. 7I-J, S5C).

### NPs delivered siLCN2 to the tumor site and maintained local concentrations

With the promising in vitro results above, we finally evaluated whether this RNAi nanoplatform could effectively deliver siRNA into tumor tissues to silence LCN2 expression and inhibit tumor progression. The pharmacokinetics were first examined by intravenous injection of NPs loaded with Cy5-siLCN2 into healthy mice (1 nmol siLCN2 dose per mouse).

**Fig. 8:**
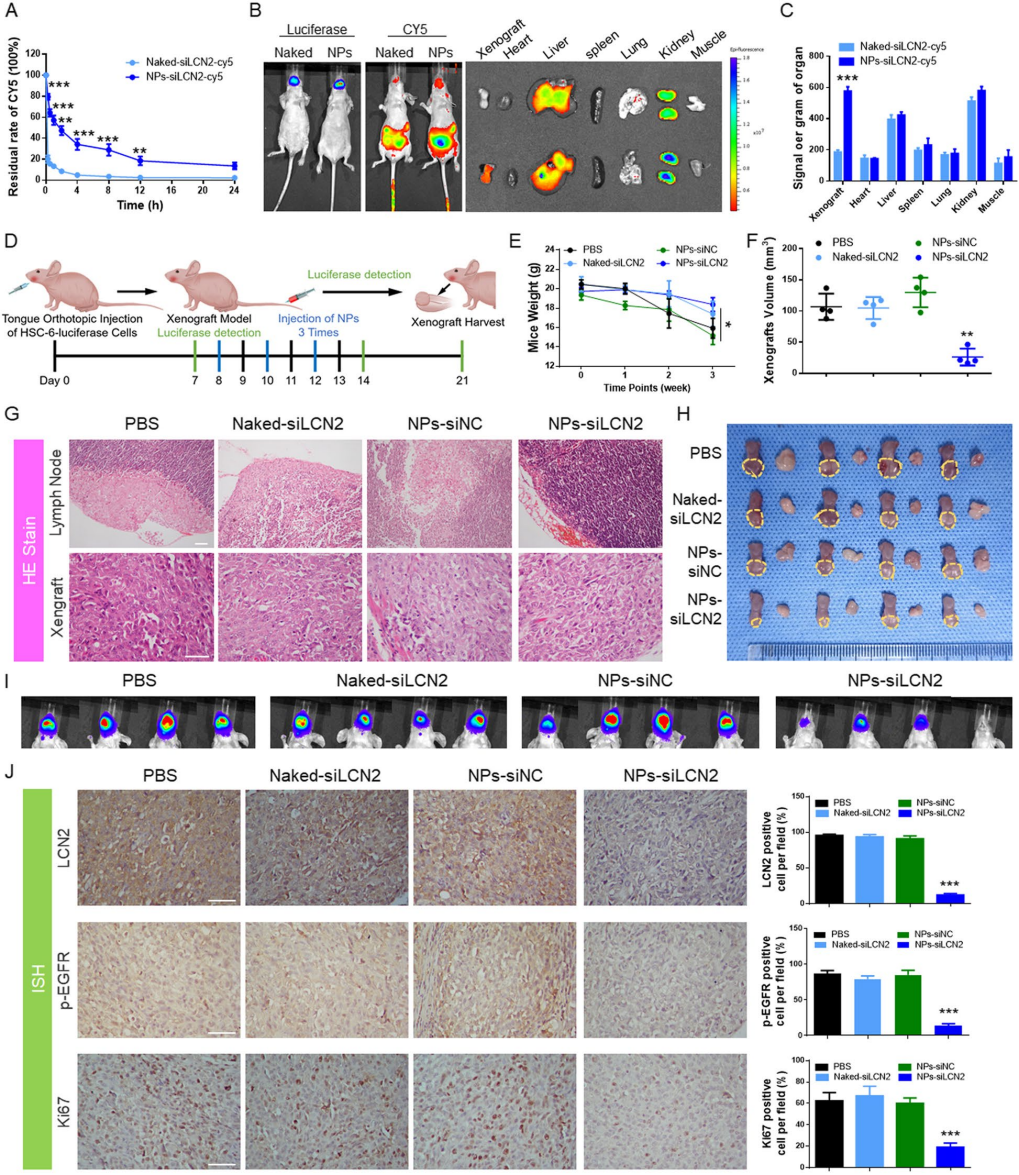
**A** The pharmacokinetics study found that nanoparticle-encapsulated siLCN2 could remain in the body for a longer period than naked siLCN2. **B** After the tongue orthotopic xenograft model was established, nanoparticles with encapsulated siLCN2-Cy5 were injected into the tail vein, and the mice were assessed 24 h later. In situ tumors of the tongue in the nanoparticle-encapsulated group still contained residual nanoparticles, while in the naked group, residual nanoparticles accumulated in the liver and kidneys. **C** Nanoparticles with encapsulated siLCN2-Cy5 were injected through the tail vein. After 24 h, the internal organs were weighed and homogenized to detect the Cy5 content in the organs. The Cy5 content in transplanted tumors was significantly higher in the nanoparticle group than in the naked siLCN2 group. **D** Schematic diagram of mouse tongue orthotopic tumor model construction, in vivo imaging observation, nanoparticle injection and material sampling. **E** The change in mouse weight. The NPs-siLCN2 group exhibited the smallest weight change, suggesting that the tumors were smaller and that their feeding was not affected. **F** Comparison of the mice. The nano-siLCN2 group had the smallest tumor weight, suggesting that the tumors were smaller. **G** HE staining of xenografts and cervical lymph nodes in the four groups. Lymph node metastasis was observed in the PBS, Naked-siLCN2 and NPs-siNC groups, but no lymph node metastasis was observed in the NPs-siLCN2 group. **H** and **I** Anatomical images and final IVIS images of orthotopic tumors and lymph nodes in the mouse tongue. In the nano-siLCN2 group, the tumor volume was significantly reduced, and no lymph node metastasis occurred. (Scale bar: 50 μm). **J** Immunohistochemical staining of tumors for LCN2, p-EGFR and Ki67. The results showed that the expression levels of LCN2, p-EGFR and Ki67 were significantly decreased in the NPs-siLCN2 group, while there was no significant difference in the other three groups. (Scale bar: 50 μm)

Due to protection by the PEG outer layer, the NPs remained stable in the mouse body for a long time (Fig. 8A). Both NPs displayed longer blood circulation than naked siLCN2 from the first 2 h, and more than 10% of injected NPs remained in the blood at 8 h postinjection. Subsequently, orthotopic tongue xenograft models of OSCC were constructed by CAL-27 luciferase labeling in nude mice, and NPs containing siLCN2-Cy5 were administered. The NPs successfully and accurately delivered siLCN2-Cy5 to the tumor area (Fig. 8B), which was also verified by homogenized detection in various tumor tissues and organs (Fig. 8C).

### NP-mediated delivery of siLCN2 inhibited cervical lymph node metastasis of OSCC tongue orthotopic xenografts

With promising in vivo delivery results, we intravenously administered the NPs to orthotopic tumor-bearing mice every two days at a 1 nmol siLCN2 dose per mouse (*n* = 4) on the 7th day of tumor formation when luciferase luminescence was detected (Fig. 8D). Tumor growth and metastasis were observed weekly with a live-animal imaging system (IVIS). At the third (21 day) observation, we found that cervical lymph node metastasis had occurred in the PBS group, naked siRNA group and NPs-siNC group but not in the NPs-siLCN2 group (Figs. 8I, S6A). The mouse weight significantly decreased with tumor growth. Therefore, we terminated the experiment at that time. The results showed that after delivery of siLCN2, the rate at which the mice lost weight was significantly slower than that of the other groups (Fig. 8E), as the tumors of the mice were the smallest (Fig. 8F). This may be because tumor growth on the tongue affected the ability of the mice to eat, causing weight loss. Tongue xenografts and cervical lymph nodes were harvested (Fig. 8H) for paraffin and histological detection. No lymph node metastasis in mice was observed in the NPs-siLCN2 group (Fig. 8G), indicating that tumor growth and metastasis were effectively suppressed. Further immunohistochemical staining (Fig. 8J) confirmed the inhibition of LCN2 expression in the NPs-siLCN2 group, and the levels of p-EGFR and the tumor proliferation index Ki67 were also decreased. This is consistent with the results of our previous in vitro studies.

### NP-mediated delivery of siLCN2 inhibited EGFR-resistant OSCC PDX progression

**Fig. 9:**
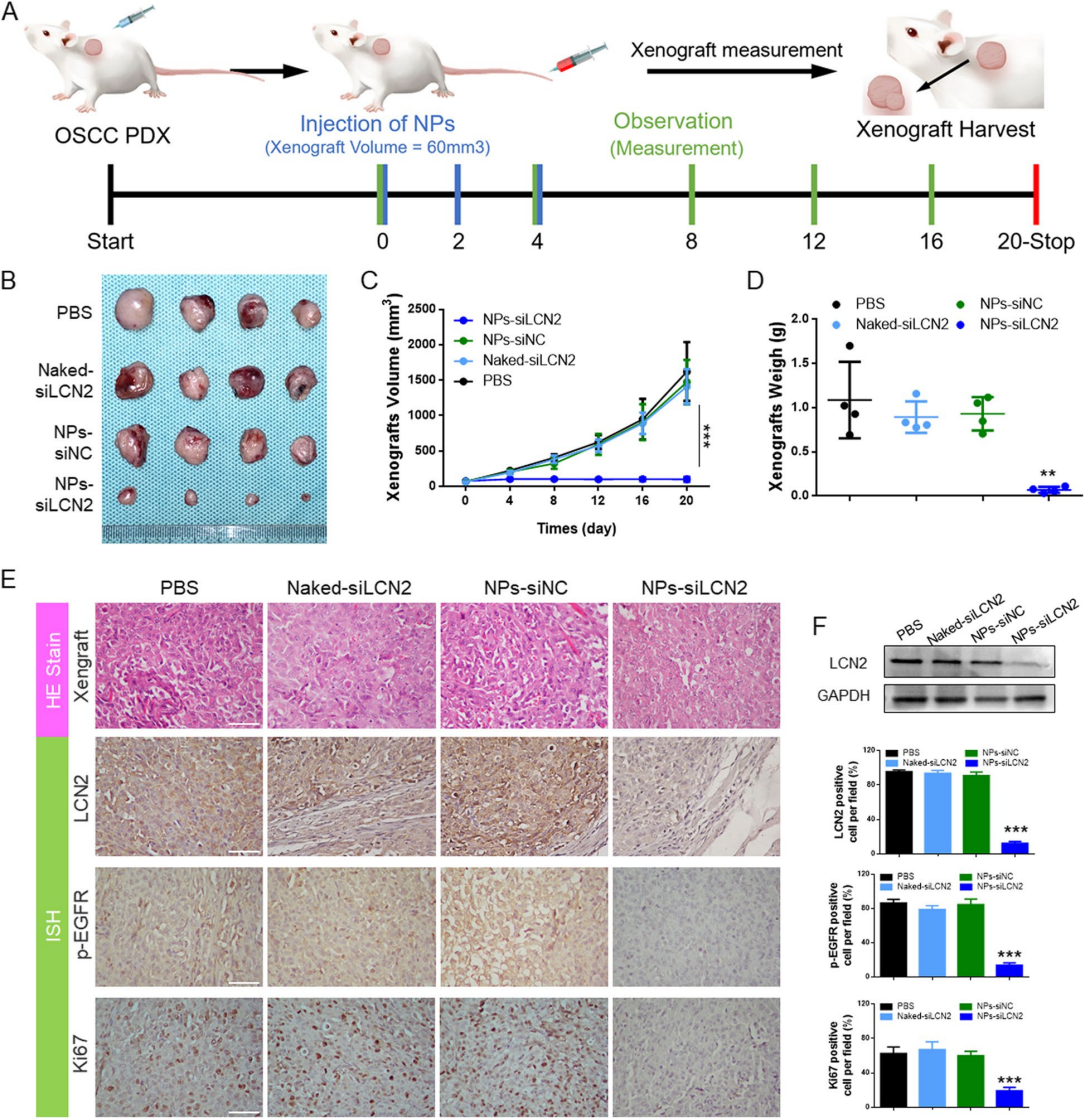
**A** Schematic diagram of the treatment of an OSCC PDX model. Three cycles of intravenous injection of NPs and 6 observations were applied when the tumor volume was approximately 60 mm^3^. **B** Photographs of collected xenografts on Day 20. Among the groups, the NPs-siLCN2 group displayed the smallest xenografts. **C** Tumor proliferation in each group. The inhibition of tumor growth was significant in the NPs-siLCN2 group compared to that in the other groups. **D** Comparison of tumor weight on the last day (Day 20). **E** Histological detection of HE, LCN2, p-EGFR and Ki67 in the tumor tissues; the results suggested that when LCN2 was inhibited, p-EGFR and Ki67 expression was reduced. (Scale bar: 50 μm). **F** Immunoblot detection of tumor homogenates. The results showed that LCN2 expression was downregulated in tumors from the nano-siLCN2 group

To further evaluate the LCN2 target for EGFR intervention in the PDX model, EGFR-positive and EGFR-resistant OSCC samples were selected. After the PDX models were established, NPs were then intravenously administered to the tumor-bearing mice every two days at a 1 nmol siRNA dose per mouse (Fig. 9A). After 3 consecutive injections, tumor growth was significantly inhibited starting on the 8th day. Tumor volumes in the NPs-siLCN2 group remained stable within 20 days. In contrast, the control group showed a dramatic increase over 20 days of observation (Fig. 9B-D). The better anticancer effect of NPs-siLCN2 was further demonstrated by histological analysis (Fig. 9E), in which the NPs were the most effective in inhibiting tumor proliferation, silencing LCN2 expression, and suppressing p-EGFR and Ki67 expression in the tumor tissues. Meanwhile, tumor homogenate experiments also confirmed that LCN2 was successfully inhibited by NPs in this process (Fig. 9F).

Moreover, it is noted that the administration of NPs does not influence the mouse weight in subcutaneous and orthotopic tumor models (Fig. S6E and S7D), implying the low in vivo toxicity of NPs-siLCN2. Moreover, mouse peripheral blood was harvested for biochemical index detection. All of the detected biochemical indicators (ALT, AST, ALB, ALP, BUN, and CREA) were within the normal range (Figs. S6C and S7C). Histological analysis of the mouse organs showed no significant difference in HE staining between the groups (Figs. S6B and S7B). The preclinical translational application showed the feasibility and safety of siLCN2 delivered by a nanomaterial to inhibit the progression of OSCC.

## Discussions

In this study, we found that EGFR activation is regulated by LCN2 through high-throughput sequencing and bioinformatics analysis. Although the sequencing results suggest that there are 3 genes as candidates, we selected LCN2 for the following exploration. PRAL is a lncRNA related to the regulation of P53, is mostly expressed at low levels in tumors and is an important tumor suppressor gene [22]. TNFSF18 is involved in the functional regulation of immune cells in tumors, especially T cells, B cells, macrophages and dendritic cells, while it has low or no expression in tumor cells [23]. Our qPCR results also revealed that the other two genes were not suitable for further study in this research.

LCN2 is also known as neutrophil gelatinase-associated lipoprotein (NGAL), which belongs to the lipocalin superfamily that was first identified by Hraba-Renevey [24]. It is involved in the regulation of a variety of tumors and is considered to be an independent predictor of tumor proliferation, metastasis potential, and differentiation [17–19, 25–27]. In terms of its mechanism, various theories have been proposed; these theories involve the ERK1/2 pathway [28, 29], regulation of the loss of E-cadherin [30, 31], formation of a complex with MMP-9 [32, 33], promotion of E-cadherin activity by changing its sublocalization [34], promotion of iron absorption [35] and promotion of epithelial to mesenchymal transition (EMT) [36, 37].

We identified LCN2 as an important regulator of OSCC metastasis and proliferation and revealed its regulatory role in EGFR. Inhibition of LCN2 expression in OSCC can suppress the activation of EGFR and its downstream MEK/ERK pathway. The reason is that LCN2 promotes the recycling of EGFR by mutual binding. Normally, the activation of EGFR by phosphorylation is strictly regulated by the dissociation and transport of members of the EGFR endocytic pathway, which controls EGFR signaling quality, intensity and duration [38, 39]. Abnormal EGFR activation in tumors may be due to abnormalities in endocytosis and transport, which cause changes in EGFR circulation [40]. When the expression of molecules that regulate EGFR endophagocytosis circulation is significantly increased, the duration of EGFR activation can be prolonged, with the relocation of EGFR and adhesion receptors at specific membrane sites, thereby promoting the metastasis and invasion of cancer cells [41]. For example, the increase in SYNJ2 in breast cancer promotes the recycling of EGFR, stimulating cell movement and tumor formation [42]. We found that LCN2 rapidly colocalized with EGFR and accumulated in the endoplasmic reticulum to assist in the recycling of EGFR after TGF-α was used to activate EGFR in OSCC. The results of IF analysis confirmed that the colocalization of LCN2 and EGFR was greater in the LCN2 overexpression group than in the control group. Immunoblot analysis clearly showed that EGFR was activated faster and that its activation lasted longer with LCN2 overexpression. Hence, LCN2 binds EGFR, assists EGFR in responding more quickly to a greater extent, activates the downstream MEK/ERK pathway, and promotes the EGFR recycling process.

Based on these results, LCN2 can be used as a blocker of EGFR and a new target to inhibit the metastasis of OSCC. However, there is currently a lack of clinically available blockers of LCN2. The direct delivery of naked siRNA did not have ideal effects due to its fast glomerular filtration rate and short half-life [43]. Nanoparticles (NPs) have enhanced permeability and retention (EPR) effects [44], do not easily pass through blood vessels in healthy tissues and are not cleared by the kidneys. However, they can be released from blood vessels in solid tumors due to blood vessel dysfunction, large vascular wall gaps, and poor structural integrity [45, 46]. NPs are stable in blood circulation and normal physiological tissues, but they quickly respond to the microenvironment and release their cargo once they reach the tumor site, increasing the local drug concentration and achieving precise delivery of the drug [47, 48]. Based on the tumor microenvironment, tumor characteristics and our previous work [49], we believe that the reduction-responsive nanoplatform is conducive to the delivery of LCN2-siRNA. Herein, we designed PEG-SS-PLGA for the encapsulation and delivery of siLCN2. Silencing LCN2 by NPs exerted a significant inhibitory effect on the metastasis of the OSCC orthotopic xenograft model with little toxicity. At the same time, it also limited OSCC PDX proliferation. From the results, the mice showed better physical condition in the NPs-siLCN2 group, suggesting that the application of nanoparticles that encapsulate LCN2 siRNA to inhibit the metastasis of OSCC has good application prospects.

## Conclusion

In summary, we have identified a key regulator, LCN2, from OSCC EGFR-resistant cell lines and metastatic patients. Downregulation of LCN2 inhibits the metastasis and proliferation of OSCC through EGFR activation and recycling. As a proof-of-principle study, we translated this mechanism to the treatment of OSCC tongue orthotopic xenografts and EGFR-positive PDX models by NP-delivered siLCN2, which effectively inhibited tumor progression and cervical lymphatic metastasis. Taken together, the results of this study may provide a new target for OSCC treatment. As a next step, further research will expand the scope of application of this study's conclusions, such as in the multidrug-combination treatment of OSCC.

## Supplementary Information

**Additional file 1: Table S1.** Basic data of sequenced patients. **Table S2.** High throughput sequencing results – Differential gene analysis. **Table S3.** Association of Lymph node metastasis in OSCC tongue xenografts.

**Additional file 2: Figure S1.** qPCR verified that the expression of three genes was increased in the process of tumor metastasis and EGFR drug resistance in CAL-27, HN-6 and ER-resistant strains. **Figure S2.** A. Cell scratch images of CAL-27ER and HN-6ER cells in the LCN2-inhibited groups; wound healing speeds were decreased. B. Cell scratch images of CAL-27 and HN-6 cells in the LCN2-overexpressing groups; the wound healing speeds were increased. C. Cell colony formation images for CAL-27ER and HN-6ER cells in the LCN2-inhibited groups. The colony formation of OSCC cells decreased significantly. D. Cell colony formation images for CAL-27 and HN-6 cells in the LCN2-overexpressing groups. The colony formation of OSCC cells increased significantly. **Figure S3.** A. List of LCN2-interacting proteins detected by LCN2 protein profiling. B. EGFR protein peak map. **Figure S4.** A. After HEK293T cells were cotransfected with LCN2-GFP and EGFR-mCH, EGFR signaling activation by TGF-α stimulation was observed. Scale bar: 20 μM. B. The membrane EGFR signal was significantly enhanced by approximately 2-fold in LCN2-ov cells, and 1/2 in siLCN2 cells before and after TNF-α-stimulated. **Figure S5.** A. When nanoparticles loaded with siLCN2-Cy5 were added to stably transfected HN-6-Endo14-GFP cells, the nanoparticles entered the cells after 4 h, were completely released from the endosomes in 6 h, and filled the entire cytoplasm after 8 h. B. The cell scratch images for CAL-27ER and HN-6ER by NP delivery; wound healing speeds were decreased in the NPs-siLCN2 groups. C. Cell colony formation images for CAL-27ER and HN-6ER cells after NP delivery. The colony formation of OSCC cells decreased in the NPs-siLCN2 groups. **Figure S6.** A. IVIS images of orthotopic tumors and lymph nodes in the mouse tongue. In the nano-siLCN2 group, the tumor volume was significantly reduced, and no lymph node metastasis occurred. B. HE staining of the organs (heart, liver, spleen, lung and kidneys) in each group. The results showed that the application of nanoparticles did not cause obvious damage to the organs of the mice, and there was no significant difference in the staining results between the groups. C. The detection of biochemical indexes in the serum of mice in each group. The results of ALT, AST, ALB, ALP, BUN, and CREA quantification showed no significant difference between the groups, suggesting that the application of the nanoparticles did not cause obvious damage to the mice. **Figure S7.** A. Tumor image and histological analysis of HE, LCN2, p-EGFR and Ki67 from the OSCC tissue donor. B. Histological staining of sections of major organs from PDX tumor-bearing mice in different treatment groups. C. Serum levels of alanine aminotransferase (ALT), aspartate aminotransferase (AST), blood urine nitrogen (BUN), alkaline phosphatase (ALP), serum albumin (ALB), and creatinine (CREA) after treatments. D. NPs did not affect the weight of mice during the treatment.

**Additional file 3. **

**Additional file 4. **

## Data Availability

The datasets used for the current study are available from the corresponding author on reasonable request. All data generated or analyzed during this study are included in this published article or its supplementary information files (Figs. S1, S2, S3, S4, S5 and S6).

## Abbreviations

EGFR: Epidermal growth factor receptor
OSCC: Oral squamous cell carcinoma
LCN2: Lipocalin 2
NP: Nanoparticle
siLCN2: LCN2 siRNA
PDX: Patient-derived xenograft
OS: Overall survival
DFS: Disease-free survival
IF: Immunofluorescence
EMT: Epithelial to mesenchymal transition
EPR: Enhanced permeation and retention
