# Correction: Silencing LCN2 suppresses oral squamous cell carcinoma progression by reducing EGFR signal activation and recycling

**DOI:** 10.1186/s13046-023-02679-0

**Published:** 2023-04-27

**Authors:** Zixian Huang, Xi Rui, Chen Yi, Yongju Chen, Rui Chen, Yancan Liang, Yan Wang, Weicheng Yao, Xiaoding Xu, Zhiquan Huang

**Affiliations:** 1 Department of Oral and Maxillofacial Surgery, Sun Yat-Sen Memorial Hospital, Sun Yat-Sen University, Guangzhou, Guangdong China; 2 Guangdong Provincial Key Laboratory of Malignant Tumor Epigenetics and Gene Regulation, Medical Research Center, Guangdong-Hong Kong Joint Laboratory for RNA Medicine, Sun Yat-Sen Memorial Hospital, Sun Yat-Sen University, Guangzhou, China; 3 Hospital of Stomatology, The First Afliated Hospital, Jinan University, Guangzhou, China; 4 Nanhai Translational Innovation Center of Precision Immunology, Sun Yat-Sen Memorial Hospital, Foshan, 528200 China; 5 Guanghua School of Stomatology, Hospital of Stomatology, Sun Yat-Sen University, Guangzhou, Guangdong China; 6 Department of Stomatology, Sun Yat-Sen Memorial Hospital, Sun Yat-Sen University, Guangzhou, Guangdong China

**Correction: *J Exp Clin Cancer Res *42**, **60 (2023)**

10.1186/s13046-023-02618-z

Following publication of the original article [1], an error was identified in Fig. 3, specifically:

• Figure 3D and 3L – images overlap

Correct figure is presented below:

**Fig. 3:**
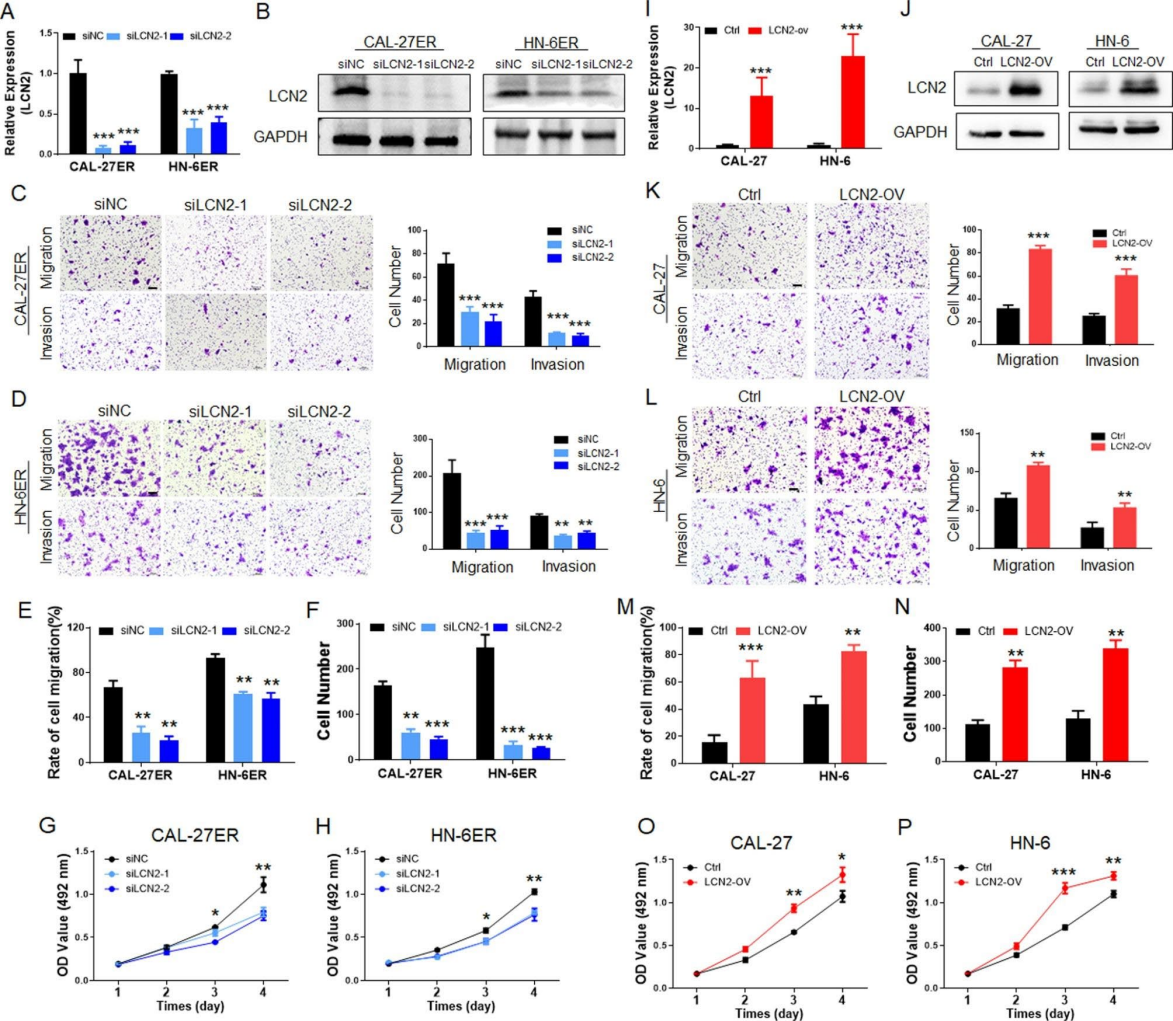
**A** PCR detection confrmed that LCN2 was successfully inhibited in the EGFR-resistant OSCC cell lines. **B** Western blotting confrmed that LCN2 was successfully inhibited in CAL-27ER and HN-6ER cells. **C** and **D** After the expression of LCN2 was downregulated, the migration and invasion of CAL-27ER cells were signifcantly inhibited, and the cells that passed through the upper chamber of the Transwell were signifcantly reduced. (Scale bar: 100 μm). **E** The scratch test showed that LCN2 was downregulated, the migration function of CAL-27ER cells was signifcantly downregulated, and the scratch healing speed (recovery rate) was decreased. **F** The cell colony formation test showed that after inhibiting LCN2 in ER-resistant cells, the colony formation of OSCC cells decreased signifcantly. **G** and **H** Inhibiting the expression of LCN2 signifcantly decreased the proliferation ability of OSCC cells, and the CCK-8 results were lower than those of the control group. **I** PCR detection confrmed that LCN2 was successfully overexpressed in wild-type CAL-27 and HN-6 cells. **J** Western blotting showed that LCN2 was successfully overexpressed in wild-type CAL-27 and HN-6 cells. **K** and **L** After overexpression of LCN2, the migration and invasion of CAL-27 cells were signifcantly upregulated, and the number of cells that passed through the upper chamber of the Transwell was signifcantly increased. (Scale bar: 100 μm). **M** After the scratch experiment confrmed that LCN2 was overexpressed, the migration ability of CAL-27 cells was signifcantly upregulated, and the scratch healing speed was increased. **N** The cell colony formation assay showed that the colony formation of OSCC cells increased signifcantly when LCN2 was overexpressed. **O** and **P** Upregulation of LCN2 in OSCC cells signifcantly increased their proliferation abilities, and the CCK-8 values were higher than those of the control group
