# Supplementary material for: Silencing LCN2 suppresses oral squamous cell carcinoma progression by reducing EGFR signal activation and recycling
Source: J Exp Clin Cancer Res. 2023 Mar 11;42:60. doi: 10.1186/s13046-023-02618-z (PMC10007849; doi:10.1186/s13046-023-02618-z)
Supplement: Supplementary file 1 — Additional file 1: Table S1. Basic data of sequenced patients. Table S2. High throughput sequencing results – Differential gene analysis. Table S3. Association of Lymph node metastasis in OSCC tongue xenografts. [file 13046_2023_2618_MOESM1_ESM.docx]

**Table S1. Basic data of sequenced patients**

| patients | Gender | Age | Tumor site | Differentiation | Lymphatic metastatic |
| --- | --- | --- | --- | --- | --- |
| 1 | Male | 46 | Right tougue | High | Yes |
| 2 | Female | 52 | Left tougue | Moderate | Yes |
| 3 | Male | 56 | Right tougue | Moderate | No |
| 4 | Female | 43 | Right tougue | High | No |

# Table S2. High throughput sequencing results – Differential gene analysis

| Gene name | Log2(Metastatic  Yes vs No) | P value | Log2 (CAL27  ER vs wt) | P value | Log2 (HN-6  ER vs wt) | P value |
| --- | --- | --- | --- | --- | --- | --- |
| LCN2 | 1.81 | <0.001 | 1.57 | <0.001 | 1.40 | <0.001 |
| PRAL | 1.28 | <0.001 | 4.09 | <0.001 | 1.20 | <0.05 |
| TNFSF18 | 3.23 | <0.001 | 4.01 | <0.001 | 1.28 | <0.001 |
| SHC3 | -2.40 | <0.001 | -1.05 | <0.05 | -1.51 | <0.01 |

**Table S3 Association of Lymph node metastasis in OSCC tongue xenografts**

| Group | Lymph node metastasis | | P Value |
| --- | --- | --- | --- |
|  | Yes | No |  |
| 1 |  |  |  |
| Ctrl-OV | 2 | 2 |  |
| LCN2-OV | 4 | 0 | 0.429 |
| 2 |  |  |  |
| Ctrl-sh | 2 | 2 |  |
| shLCN2 | 0 | 4 | 0.429 |
| 3 |  |  |  |
| shLCN2 | 0 | 4 |  |
| LCN2-OV | 4 | 0 | 0.029 |
| 4 |  |  |  |
| Ctrl-sh | 2 | 2 |  |
| Ctrl-OV | 2 | 2 | 1.000 |
|  | | |  |
