# Supplementary material for: Silencing LCN2 suppresses oral squamous cell carcinoma progression by reducing EGFR signal activation and recycling
Source: J Exp Clin Cancer Res. 2023 Mar 11;42:60. doi: 10.1186/s13046-023-02618-z (PMC10007849; doi:10.1186/s13046-023-02618-z)
Supplement: Supplementary file 2 — Additional file 2: Figure S1. qPCR verified that the expression of three genes was increased in the process of tumor metastasis and EGFR drug resistance in CAL-27, HN-6 and ER-resistant strains. Figure S2. A. Cell scratch images of CAL-27ER and HN-6ER cells in the LCN2-inhibited groups; wound healing speeds were decreased. B. Cell scratch images of CAL-27 and HN-6 cells in the LCN2-overexpressing groups; the wound healing speeds were increased. C. Cell colony formation images for CAL-27ER and HN-6ER cells in the LCN2-inhibited groups. The colony formation of OSCC cells decreased significantly. D. Cell colony formation images for CAL-27 and HN-6 cells in the LCN2-overexpressing groups. The colony formation of OSCC cells increased significantly. Figure S3. A. List of LCN2-interacting proteins detected by LCN2 protein profiling. B. EGFR protein peak map. Figure S4. A. After HEK293T cells were cotransfected with LCN2-GFP and EGFR-mCH, EGFR signaling activation by TGF-α stimulation was observed. Scale bar: 20 μM. B. The membrane EGFR signal was significantly enhanced by approximately 2-fold in LCN2-ov cells, and 1/2 in siLCN2 cells before and after TNF-α-stimulated. Figure S5. A. When nanoparticles loaded with siLCN2-Cy5 were added to stably transfected HN-6-Endo14-GFP cells, the nanoparticles entered the cells after 4 h, were completely released from the endosomes in 6 h, and filled the entire cytoplasm after 8 h. B. The cell scratch images for CAL-27ER and HN-6ER by NP delivery; wound healing speeds were decreased in the NPs-siLCN2 groups. C. Cell colony formation images for CAL-27ER and HN-6ER cells after NP delivery. The colony formation of OSCC cells decreased in the NPs-siLCN2 groups. Figure S6. A. IVIS images of orthotopic tumors and lymph nodes in the mouse tongue. In the nano-siLCN2 group, the tumor volume was significantly reduced, and no lymph node metastasis occurred. B. HE staining of the organs (heart, liver, spleen, lung and kidneys) in each group. The res [file 13046_2023_2618_MOESM2_ESM.pdf]

Silencing LCN2 suppresses oral squamous cell carcinoma progression by reducing EGFR signal activation and recycling

Supply Figures & Figure legends

Figure S1

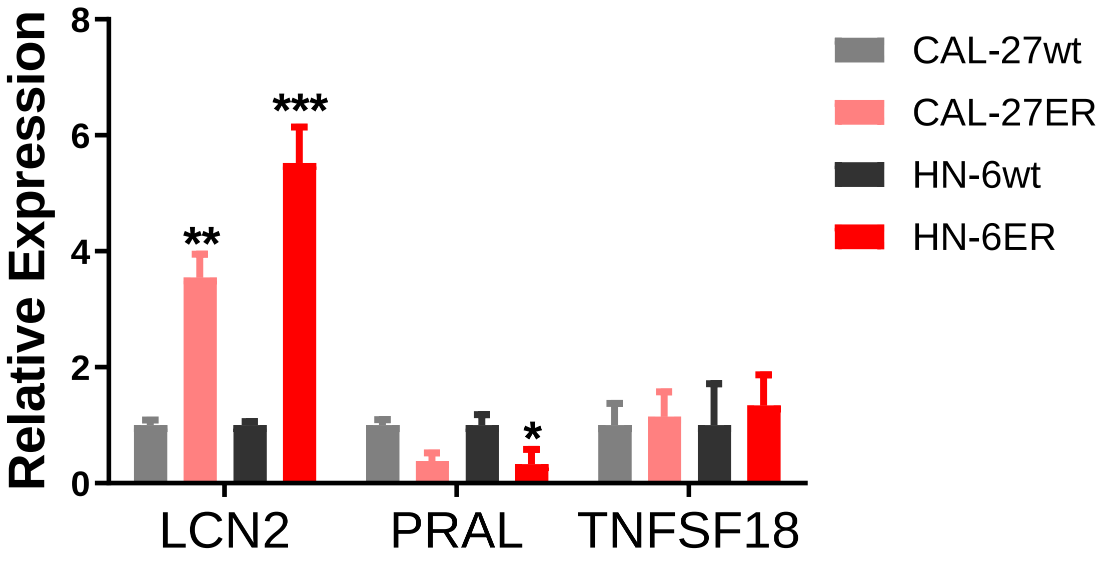

Figure S1

qPCR verified that the expression of three genes was increased in the process of tumor metastasis and EGFR drug resistance in CAL-27, HN-6 and ER-resistant strains.

Figure S2

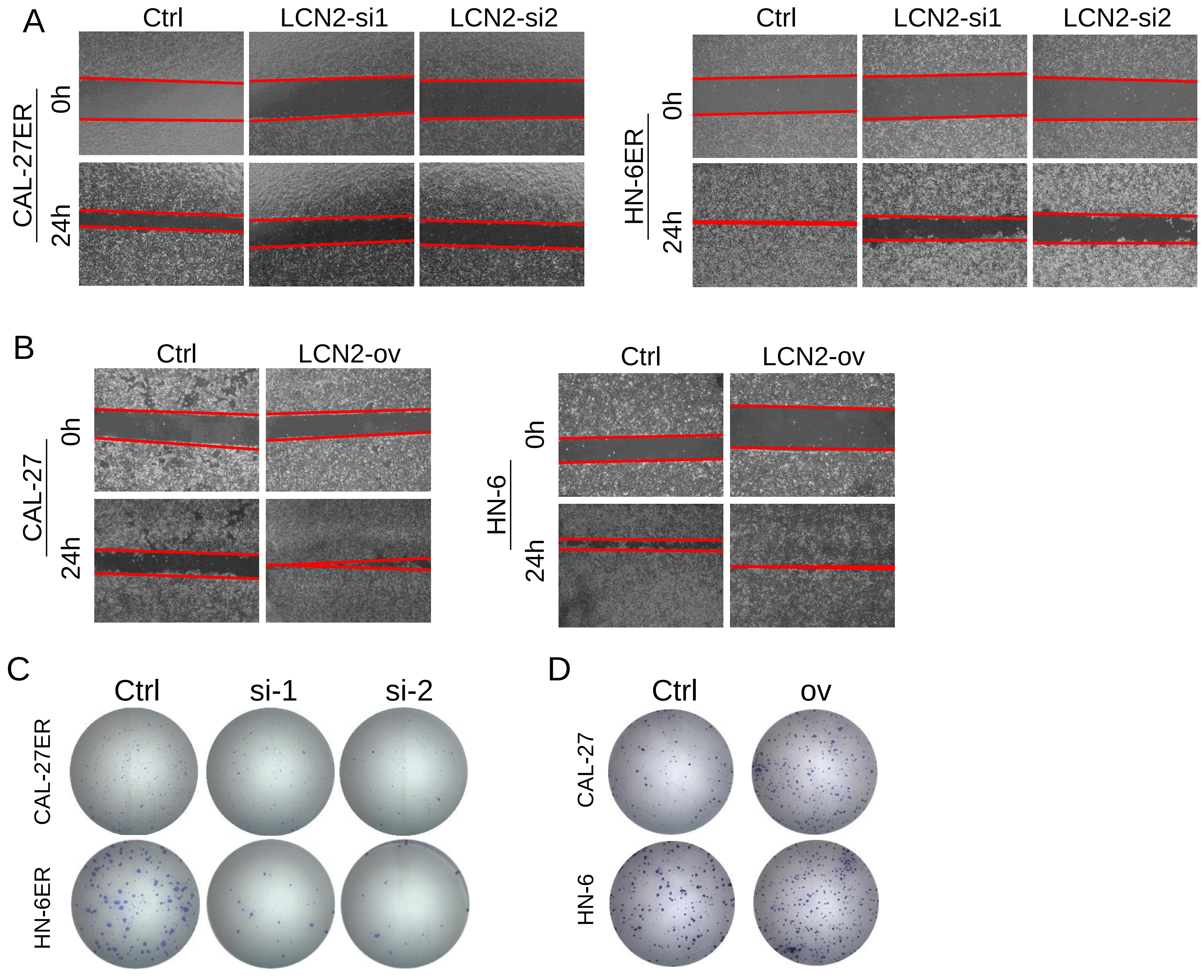

Figure S2

A. Cell scratch images of CAL-27ER and HN-6ER cells in the LCN2-inhibited groups; wound healing speeds were decreased.

B. Cell scratch images of CAL-27 and HN-6 cells in the LCN2-overexpressing groups; the wound healing speeds were increased.

C. Cell colony formation images for CAL-27ER and HN-6ER cells in the LCN2-inhibited groups. The colony formation of OSCC cells decreased significantly.

D. Cell colony formation images for CAL-27 and HN-6 cells in the LCN2-overexpressing groups. The colony formation of OSCC cells increased significantly.

Figure S3

A

Gene symbol	*Unique*	*Coverage(%)*	*Gene symbol*	*Unique*	*Coverage %*
EGFR	6	28	UBE2V1	1	7
RPS3	4	34	PSMC1	1	3
HMGB1	3	13	PSMC5	1	3
PSMD3	3	7	CALR	1	2
EEF1D	3	20	PSME3	1	4
FLNA	3	1	LTF	1	1
PSMA7	2	10	PSMD2	1	1
ZC3HAV1	2	4	PSMD4	1	3
MTDH	2	4	LGALS1	1	11
RHOC	1	6	PSMC2	1	3
UBE2I	1	8	PSMD11	1	3
SKP1	1	7			

B

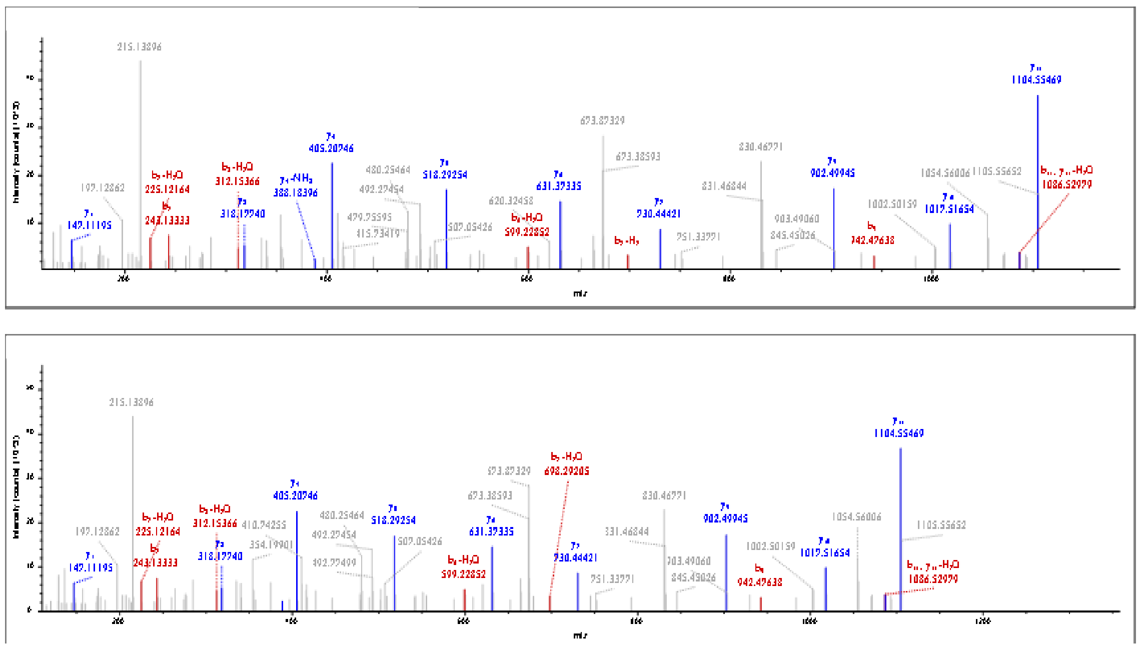

Figure S3

A. List of LCN2-interacting proteins detected by LCN2 protein profiling.

B. EGFR protein peak map.

Figure S4

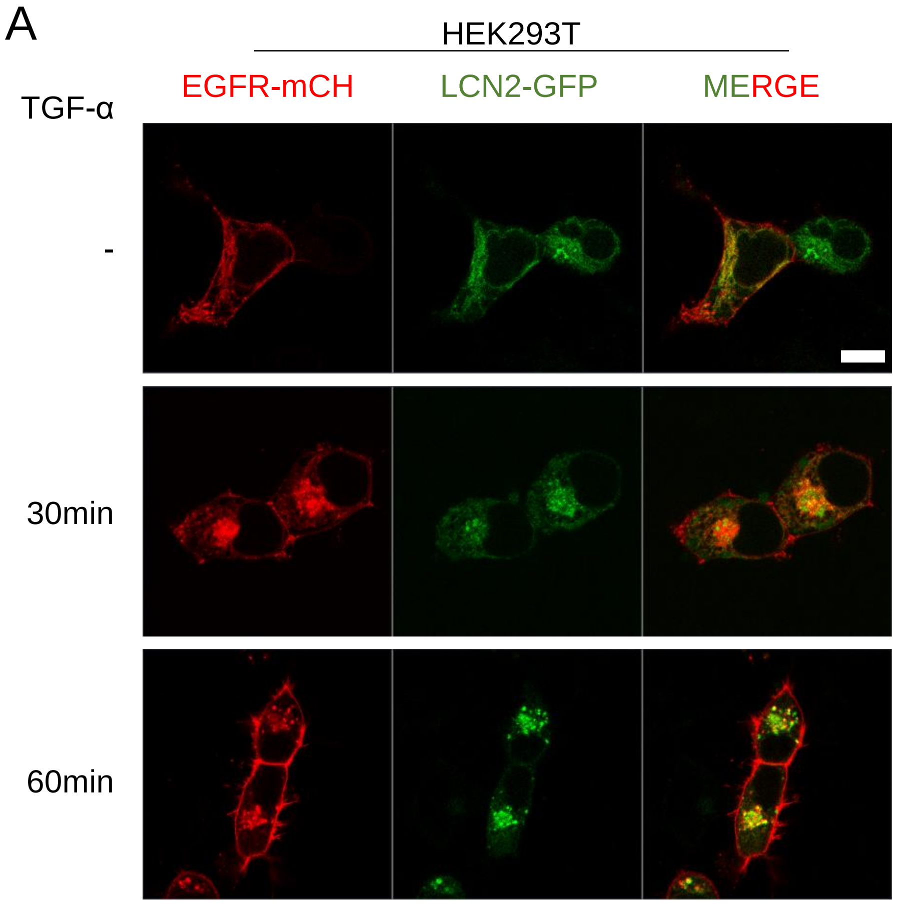

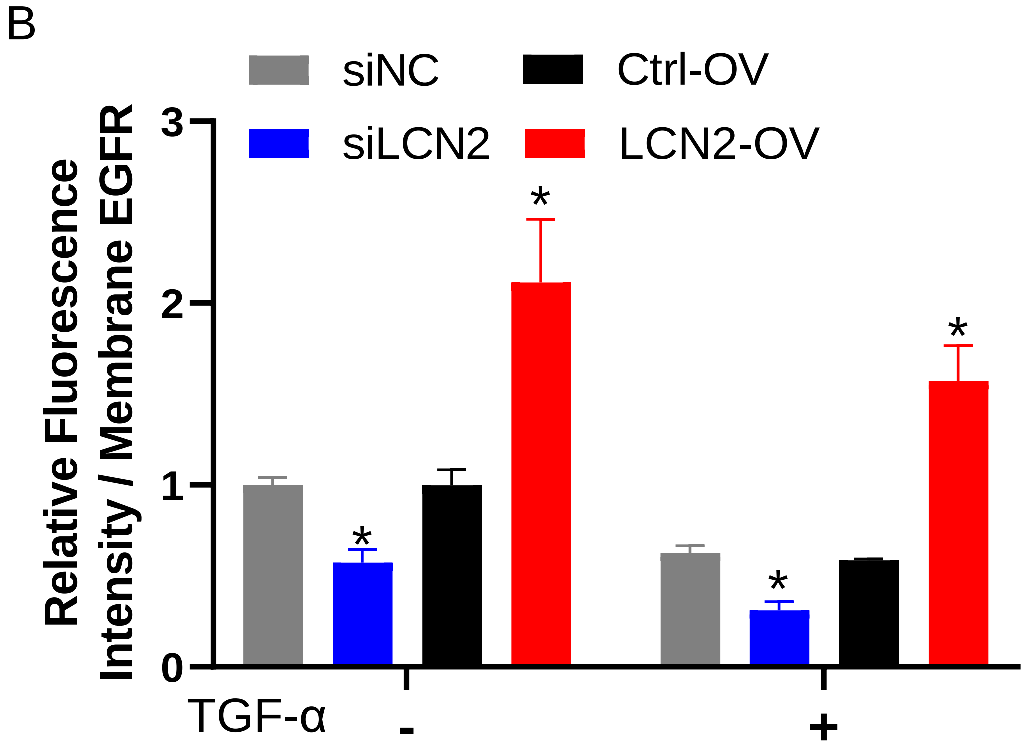

Figure S4

A. After HEK293T cells were cotransfected with LCN2-GFP and EGFR-mCH, EGFR signaling activation by TGF-α stimulation was observed. Scale bar: 20 μM.

B. The membrane EGFR signal was significantly enhanced by approximately 2-fold in LCN2-ov cells, and 1/2 in siLCN2 cells before and after TNF-α-stimulated.

Figure S5

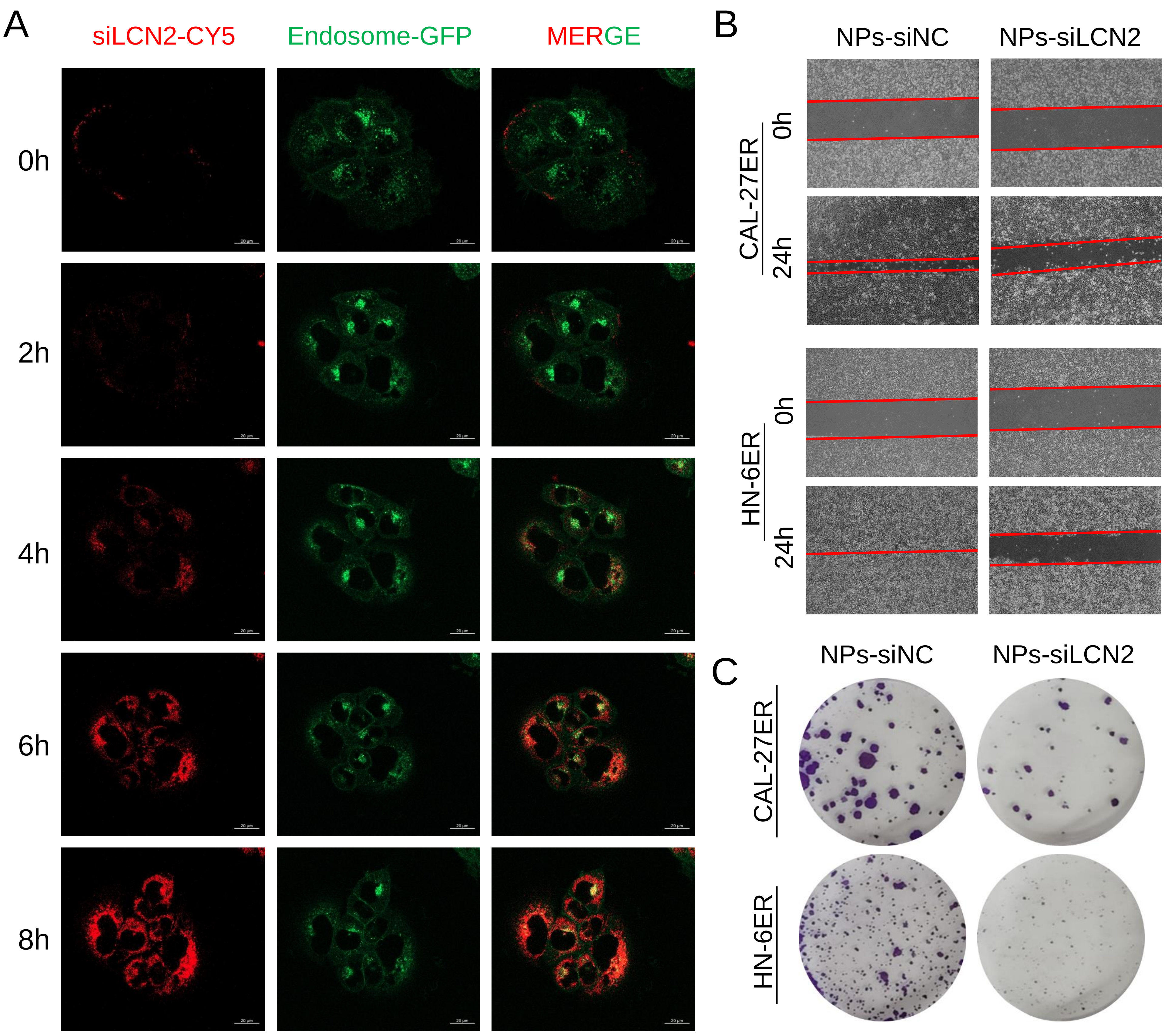

Figure S5

A. When nanoparticles loaded with siLCN2-Cy5 were added to stably transfected CAL-27-Endo14-GFP cells, the nanoparticles entered the cells after 4 h, were completely released from the endosomes in 6 h, and filled the entire cytoplasm after 8 h.

B. The cell scratch images for CAL-27ER and HN-6ER by NP delivery; wound healing speeds were decreased in the NPs-siLCN2 groups.

C. Cell colony formation images for CAL-27ER and HN-6ER cells after NP delivery. The colony formation of OSCC cells decreased in the NPs-siLCN2 groups.

Figure S6

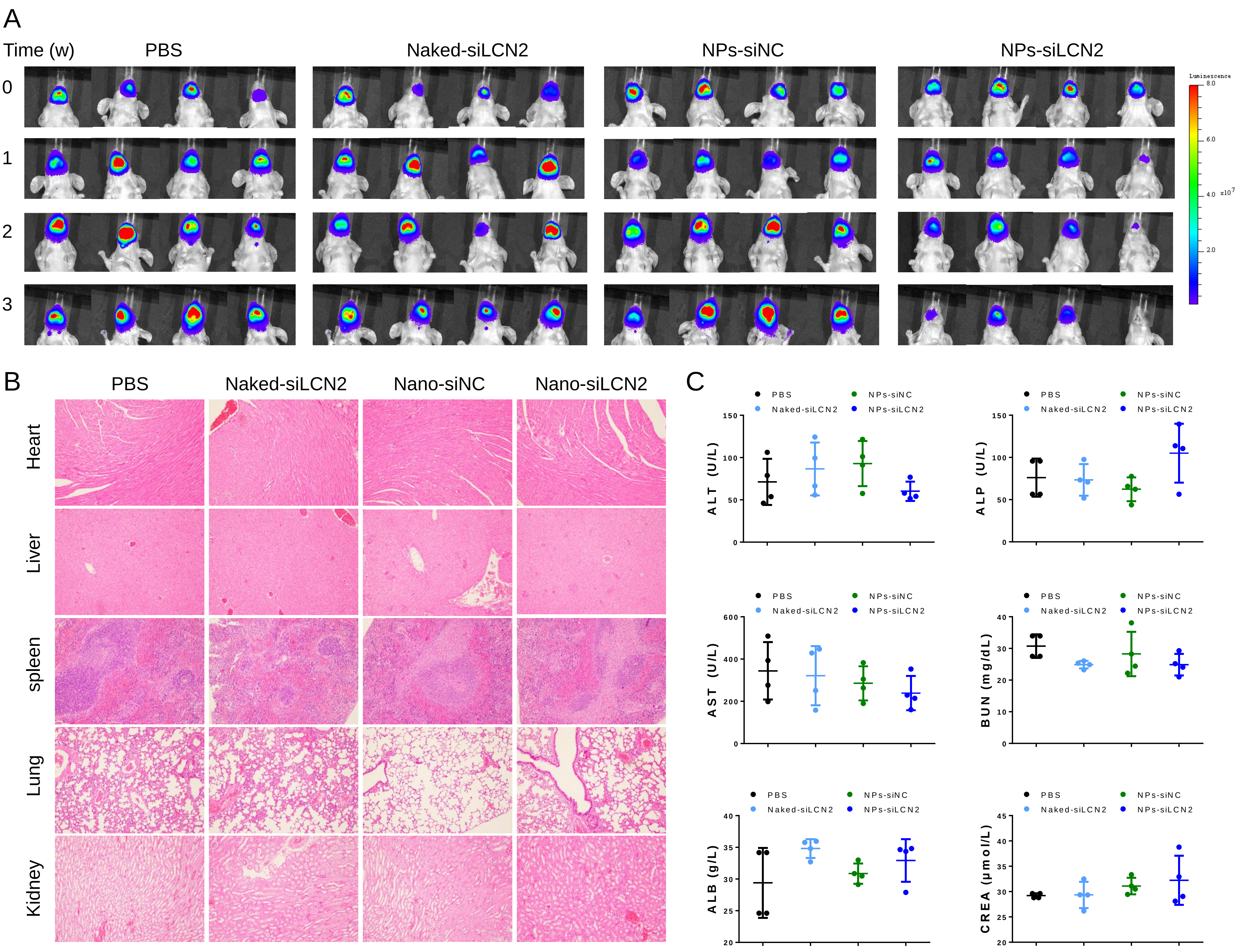

Figure S6

A. IVIS images of orthotopic tumors and lymph nodes in the mouse tongue. In the nano-siLCN2 group, the tumor volume was significantly reduced, and no lymph node metastasis occurred.

B. HE staining of the organs (heart, liver, spleen, lung and kidneys) in each group. The results showed that the application of nanoparticles did not cause obvious damage to the organs of the mice, and there was no significant difference in the staining results between the groups.

C. The detection of biochemical indexes in the serum of mice in each group. The results of ALT, AST, ALB, ALP, BUN, and CREA quantification showed no significant difference between the groups, suggesting that the application of the nanoparticles did not cause obvious damage to the mice.

Figure S7

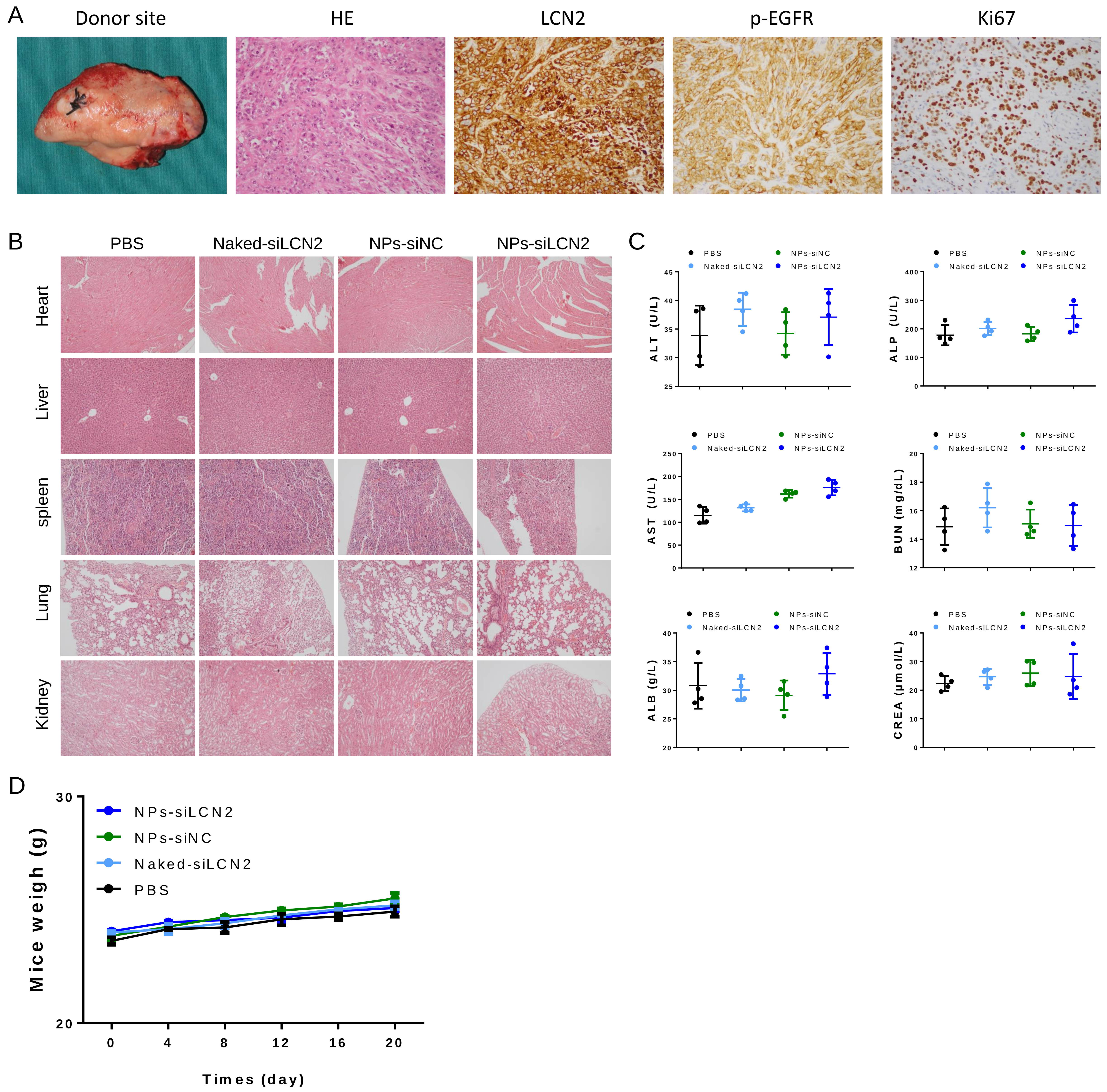

Figure S7
A. Tumor image and histological analysis of HE, LCN2, p-EGFR and Ki67 from the OSCC tissue donor.
B. Histological staining of sections of major organs from PDX tumor-bearing mice in different treatment groups.
C. Serum levels of alanine aminotransferase (ALT), aspartate aminotransferase (AST), blood urine nitrogen (BUN), alkaline phosphatase (ALP), serum albumin (ALB), and creatinine (CREA) after treatments.
D. NPs did not affect the weight of mice during the treatment.
